# Effects of MCHM on yeast metabolism

**DOI:** 10.1371/journal.pone.0223909

**Published:** 2019-10-17

**Authors:** Amaury Pupo, Kang Mo Ku, Jennifer E. G. Gallagher

**Affiliations:** 1 Department of Biology, West Virginia University, Morgantown, West Virginia, United States of America; 2 Division of Plant and Soil Sciences, West Virginia University, Morgantown, West Virginia, United States of America; 3 Department of Horticulture, College of Agriculture and Life Sciences, Chonnam National University, Gwangju, Republic of Korea; University of Florida, UNITED STATES

## Abstract

On January 2014 approximately 10,000 gallons of crude 4-Methylcyclohexanemethanol (MCHM) and propylene glycol phenol ether (PPH) were accidentally released into the Elk River, West Virginia, contaminating the tap water of around 300,000 residents. Crude MCHM is an industrial chemical used as flotation reagent to clean coal. At the time of the spill, MCHM's toxicological data were limited, an issue that has been addressed by different studies focused on understanding the immediate and long-term effects of MCHM on human health and the environment. Using *S*. *cerevisiae* as a model organism we study the effect of acute exposure to crude MCHM on metabolism. Yeasts were treated with MCHM 550 ppm in YPD for 30 minutes. Polar and lipid metabolites were extracted from cells by a chloroform-methanol-water mixture. The extracts were then analyzed by direct injection ESI-MS and by GC-MS. The metabolomics analysis was complemented with flux balance analysis simulations done with genome-scale metabolic network models (GSMNM) of MCHM treated vs non-treated control. We integrated the effect of MCHM on yeast gene expression from RNA-Seq data within these GSMNM. A total of 215 and 73 metabolites were identified by the ESI-MS and GC-MS procedures, respectively. From these 26 and 23 relevant metabolites were selected from ESI-MS and GC-MS respectively, for 49 unique compounds. MCHM induced amino acid accumulation, via its effects on amino acid metabolism, as well as a potential impairment of ribosome biogenesis. MCHM affects phospholipid biosynthesis, with a potential impact on the biophysical properties of yeast cellular membranes. The FBA simulations were able to reproduce the deleterious effect of MCHM on cellular growth and suggest that the effect of MCHM on ubiquinol:ferricytochrome c reductase reaction, caused by the under-expression of *CYT1* gene, could be the driven force behind the observed effect on yeast metabolism and growth.

## Introduction

On January 2014 approximately 10,000 gallons of crude 4-Methylcyclohexanemethanol (MCHM) and propylene glycol phenol ether were accidentally released into the Elk River, West Virginia, contaminating the tap water of around 300,000 residents [1]. Crude MCHM is an industrial chemical used as flotation reagent to clean coal [2]. More than 300 people in the affected area visited emergency departments with reports of symptoms potentially related to the spill, including mild skin, gastrointestinal and respiratory symptoms that resolved with no or minimal treatment [3]. At the time of the spill, MCHM's toxicological data were limited, an issue that have been addressed by different studies focused on understanding the immediate and long-term effects of MCHM on human health and the environment [4].

MCHM is considered a moderate-to-strong dermal irritant, causes fetal malformations in rats when orally exposed to 400 mg/kg/day [5]. The highest concentration of MCHM detected at the water treatment facility was 3.772 ppm and in treated household tap water was 0.42 ppm [6]. Crude MCHM is not a dermal irritant to humans at the concentrations in the water reported after the spill [7]. In the evaluation of different cell lines, HEK-293, HepG2, H9c2, and GT1-7 only the highest dose of MCHM (128 ppm or 1 mM) elicited a statistically significant decrease in cell viability, when compared to the control (1% DMSO) [8]. MCHM induced DNA damage-related biomarkers in human A549 cells, indicating that it is related to genotoxicity [9]. MCHM affected the larval visual-motor response in an acute developmental toxicity assay with zebrafish embryos [10]. In a limited screen, MCHM induces chemical stress related to transmembrane transport activity and oxidative stress in yeast [9].

The budding yeast *Saccharomyces cerevisiae* is one of the most intensively investigated, well-consolidated and widely used eukaryotic model organism. Its use has allowed the gain of insights in basic cellular mechanisms such as cell cycle progression, DNA replication, vesicular trafficking, protein turnover, longevity and cell death [11] or even more complex processes like neurodegenerative disorders [12]. Being among the first components of the biota to be exposed to environmental pollutants, bacteria and fungi are common model organisms for eco-toxicological assessments [13]. A number of features make *S*. *cerevisiae* an ideal model for functional toxicological studies, such as: being unicellular, the ease of genetic manipulation, availability of a huge repertoire of dedicated experimental tools, protocols, software and databases, a high degree of functional conservation with more complex eukaryotes, among others [13]. The effect of tens of pesticides has been studied in *S*. *cerevisiae* by a battery of omics approaches, including transcriptomics, chemogenomics, proteomics and metabolomics (reviewed in [13]).

Focused on the analysis of the whole repertoires of endogenous or exogenous metabolites that are present in a biological system at a given time point metabolomics serves as a link between genotype and phenotype [14,15]. Metabolomics is an extremely useful tool in the analysis of the metabolic modifications induced by potentially toxic compounds [16]. These studies include the effect different fungicides [17], Cu^2+^ exposure [18]⁠, tolerance to representative inhibitors [19], ethanol tolerance [20,21], among others.

Flux balance analysis (FBA) [22–25] with genome-scale metabolic network models (GSMNM) allows the simulation of the metabolism at a systemic level, for the understanding of diverse phenomena and making predictions [26]. There are more than twenty genome-scale metabolic network models reconstructed for *S*. *cerevisiae* to date [27]. The consensus yeast metabolic network stands out with 14 compartments, more than 3700 reactions, >2500 metabolites and >1100 genes [28].

The accuracy of FBA predictions can be improved by the integration of experimental data [26]. Several methods have been developed to this end, allowing the integration of transcriptomics data: such as E-Flux [29], omFBA [30] and transcriptional regulated flux balance analysis (TRFBA) [31]; proteomics data: GECKO (a method that enhances a genome-scale metabolic models to account for enzymes as part of reactions) [32]; and metabolomics data: unsteady-state flux balance analysis (uFBA) [33].

In the present work, we study the effect of MCHM on metabolism using yeast as a model organism, combining metabolomics tools with FBA simulations on genome-scale metabolic network models of yeast constrained by RNA-Seq data. We found that MCHM treatment altered metabolites and gene expression across metabolic pathways. Amino acid levels as well as, many amino acid precursors increased while phospholipids decreased. The increase in amino acid levels could be explained at the transcriptomic level as amino acid biosynthetic genes were upregulated. Ribosome biosynthesis genes were downregulated, which is often seen in response to stress. Several genes involved in mitochondrial function were upregulated. The role of the mitochondria in MCHM was further supported by merging the metabolomics and transcriptomics data in a flux balance analysis. This predicted that the growth inhibition of MCHM would be minimized as the concentration of D-Glucose decreased. FBA simulations suggest Ubiquinol:ferricytochrome c as the limiting reaction, which would be caused by the MCHM induced under-expression of CYT1 gene.

## Materials and methods

### MCHM treatment

Wildtype yeast from the S288c background (BY4741 strain *his3*, *ura3*, *leu2*, *met15*) [34] were grown in YPD to exponential phase (OD 0.4–0.6) then treated with crude 4-Methylcyclohexanemethanol (crude MCHM provided directly from Eastman Chemical) 550 ppm (3.9 mM) for 30 minutes or left untreated. Six independent biological replicates were done per treated and untreated group. After 30 minutes 5 optical units of cells were collected, washed with deionized water, flash-frozen in liquid nitrogen and stored at -80°C for extraction within the next 24 hours.

### Metabolites extraction

Lipid and polar metabolites were extracted with a 1:2:0.8 mixture of chloroform: MeOH: H_2_O, following a modified version of a published protocol [35]. HPLC grade chloroform and methanol were from Sigma-Aldrich. All the steps were done using glassware, to avoid polymers contamination. The extractions were performed in 15 mL Kimble^™^ Kontes^™^ KIMAX^™^ Reusable High Strength Centrifuge Tubes from Fisher Scientific. Half of the original protocol volume values were used. For extractions headed to GC-MS analysis, 50 μL of ribitol internal standard (10 mg/mL) were added. 3 mL of the polar and 3 mL of the lipid phase were collected per sample. The polar phase was dried in SpeedVac (ThermoFisher Scientific). The lipid phase was dried overnight in a fume hood. For ESI-MS experiments, but not for GC-MS, the dried polar phases were re-suspended in 500 μL of MeOH and the lipid phases were re-suspended in 500 μL 1:1 chloroform: MeOH. All extracts were stored at -20 ^o^C for analysis within 48 hours.

### ESI-MS

Samples were analyzed by direct injection of the resuspended extracts in a Thermo Fisher Scientific Q-Exactive, with an ESI (electrospray ion source), using positive and negative modes. For polar compounds in positive mode the injection speed was 10 μL/min, the scan range was 50–750 m/z, no fragmentation, 140,000 resolution, 1 microscan, AGC target 5x, maximum injection time of 100, sheath gas flow rate of 10, aux gas flow rate of 2, no sweep gas flow, spray voltage 3.60 kV, capillary temperature of 320°C, S-lens RF level 30.0. For polar compounds in the negative mode most parameters remain the same, except for spray voltage: 3.20 kV, capillary temperature: 300 ^o^C, S-lens RF level: 25.0. For lipid compounds in positive mode the following parameters were modified; scan range: 150.0–2,000.0 m/z, sheath gas flow rate: 15, aux gas flow rate: 11, spray voltage: 3.50, capillary temperature: 300°C, S-lens RF level: 25.0. For lipid compounds in negative mode the previous parameters were kept, except for the spray voltage, which was set to 3.20 kV.

50 scans were obtained per sample and later averaged with Thermo Scientific Xcalibur 2.1 SP1. Averaged spectra in the positive and negative mode were processed for polar and lipid fractions, separately with xcms 3.2.0 [36]. Peaks were identified within each spectrum using the *mass spec wavelet* method from the MassSpecWavelet 1.46.0 R package [37]. Peaks were grouped with the *Mzclust* method, followed by *groupChromPeaks*. All features were plotted visually inspected. The intensity values of each feature in each sample were obtained with the *featureValue* method as the integrated signal area for each representative peak per sample. The feature intensity and feature definition tables were saved as CSV files. Feature intensities were normalized by the total sum of the intensities of all the features detected in the corresponding spectrum (being identified or not). The normalization was done spectrum wise, so the normalized feature intensity values were a percentage of the total intensity of the spectrum of origin. Features were identified via MetaboSearch 1.2 [38], with the list comprising the average mz values for each feature as a query, with 5 ppm of error, positive or negative mode and using the four online databases available as options in the program: HMDB, Metlin, MMCD, and LipidMaps.

After the feature identification, normalized feature intensity tables (keeping only identified features) coming from the same biological replicate (both positive and negative modes from polar and lipid fractions) were merged as a single intensity table.

Features ids were confirmed by targeted MS/MS experiments, with selected features m/z values included in a target list. The isolation width in the quadrupole was 1.0 m/z and nitrogen was used as the collision gas. The fragment ions were measured in the Orbitrap with a resolution of 17,500 FWHM at 200 m/z, accumulation target 1E5, maximum fill time 60 ms and normalized collision energy of 29. The resulting fragmentation spectra were queried against Metlin and HMDB, with a mass error of 5 ppm.

Six biological replicates per group for MCHM treated and untreated controls were used. The experiment was repeated twice with consistent results. These biological replicates were not the same used in GC-MS experiments.

### GC-MS

50 μL of Methyl heptadecanoate 2 mg/mL was added as the internal standard to each lipid sample before derivatization. Lipid and polar fractions were derivatized with BSTFA [39] and MSTFA [40], respectively. For BSTFA derivatization dried extracts were treated with 200 μLL N,O-bis(trimethylsilyl)trifluoroacetamide with 1% of trimethylchlorosilane at 75°C for 30 min. For MSTFA derivatization dried extracts were treated with 50 μL methoxyamine hydrochloride (40 mg/ml in pyridine) for 90 min at 37°C, then with 100 μL MSTFA + 1% TMCS at 50°C for 20 min. Derivatized samples were analyzed using a GC-MS (Trace 1310 GC, Thermo Fisher Scientific, Waltham, MA, USA) coupled to an MS detector system (ISQ QD, Thermo Fisher Scientific, Waltham, MA, USA) and an autosampler (Triplus RSH, Thermo Fisher Scientific, Waltham, MA). A capillary column (Rxi-5Sil MS, Restek, Bellefonte, PA, USA; 30 m × 0.25 mm × 0.25 μm capillary column w/10 m Integra-Guard Column) was used to detect polar metabolites. For water-soluble metabolite analysis, after an initial temperature hold at 80°C for 2 min, the oven temperature was increased to 330°C at 15°C min^-1^ and held for 5 min. For lipid-soluble metabolite analysis, after an initial temperature hold at 150°C for 1 min, the oven temperature was increased to 320°C at 12°C min^-1^ and held for 7 min. Injector and detector temperatures were set at 250°C and 250°C, respectively. An aliquot of 1 μL was injected with the split ratio of 70:1. The helium carrier gas was kept at a constant flow rate of 1.2 mL min^-1^. The mass spectrometer was operated in positive electron impact mode (EI) at 70.0 eV ionization energy at m/z 40–500 scan range.

Peak identification and grouping, and feature intensities calculation were performed with Thermo Scientific^™^ Chromeleon^™^ (Version 7.2, Thermo Fisher Scientific, Waltham, MA, USA). Features were identified against a locally characterized set of central metabolites (targeted metabolomics), when possible. Other features were identified querying NIST database (untargeted metabolomics). Feature intensity tables were saved as CSV files, keeping only the identified features.

Features intensities from lipid and polar fractions were normalized against its corresponding internal standards (methyl heptadecanoate for lipid and ribitol for polar fractions) and then the ones coming from the same biological replicate (both lipid and polar fractions) were merged as a single intensity table.

Six biological replicates per group for MCHM treatment and untreated controls were used. The experiment was repeated three times with consistent results. These biological replicates were not the same used in ESI-MS experiments.

### Metabolomics data analysis

Feature intensity tables from ESI-MS and GC-MS were processed with MetaboAnalyst 4.0 [41] and R 3.6.1. Missing intensity values were replaced by half of the minimum positive value in the original data, before normalization. Up to 5% of the features with near-constant intensity values among the samples were filtered out. Samples were scaled by Pareto scaling. Samples were compared by univariate analysis (t-test and fold change, using R) and multivariate analysis: Principal Component Analysis (PCA), Partial Least Squares Discriminant Analysis (PLS-DA), Sparse Partial Least Squares—Discriminant Analysis (sPLS-DA), Orthogonal-Orthogonal Projections to Latent Structures Discriminant Analysis (OPLS-DA), Empirical Bayesian Analysis of Microarray (EBAM), Random Forest classification, Support Vector Machine (SVM) and Significance Analysis of Microarray (SAM), as implemented in MetaboAnalyst 4.0. For the selection of relevant metabolites, a majority vote model was built (for ESI-MS and GC-MS independently). Metabolites were selected as relevant if they were significant in at least five of the nine previously mentioned analysis. The following criteria were followed by analysis type to select the metabolites: t-test (p adjusted < 0.05), PCA (abs(PC1 loadings) > 0.1 for ESI-MS, and abs(PC1 loadings) > 0.1 OR abs(PC2 loadings) > 0.1 for GC-MS), PLS-DA (VIP component 1 > 1 for ESI-MS, and VIP component 1 > 1 OR VIP component 2 > 1 for GC-MS), sPLS-DA (abs(loadings component 1) > 0), OPLS-DA (abs(loadings component 1) > 1), EBAM, SAM, Random Forest and SVM (compounds labeled as significant within the analysis).

PLS-DA and OPLS-DA models were validated by permutations as implemented in MetaboAnalyst 4.0 [41]. Briefly, 1000 permutations were performed. In each permutation, a model was built between the data (X) and the permuted class labels (Y) using the optimal number of components determined by cross-validation for the model based on the original class assignment. For PLS-DA the separation distance based on the ratio of the between group sum of the squares and the within group sum of squares (B/W-ratio) was used for measuring class discrimination. For OPLS-DA the cross-validated R2Y and Q2 coefficients were used.

The performance of the sPLS-DA models was evaluated using leave-one-out cross-validations.

The heatmaps of the relevant compounds were done with the R package pheatmap 1.0.12.

The Pathway Analysis was performed with MetaboAnalyst 4.0 using the name of the relevant compounds from the ESI-MS and GC-MS combined. The *Saccharomyces cerevisiae* pathway library was used, as well as the hypergeometric test for the over-representation analysis and relative-betweenness centrality for the pathway topology analysis.

Some pathways were represented as Escher maps [42] with the thick and color of the edges as a function of the respective MCHM treated vs untreated control flux ratio values.

### Transcriptomics

A fraction of previously reported data was used, including only the samples with wildtype S288c (S96 *lys5)* cells in YPD treated or not with MCHM [43]. The RNA-seq of S96 was carried out on hot acid phenol extracted RNA [44]. The raw data is accessible at ttps://www.ncbi.nlm.nih.gov/geo/query/acc.cgi?acc=GSE108873, containing count data generated via Rsubread and the differential expression data generated via DESeq2. MA plot and KEGG Pathway Enrichment Analysis were done with R packages *ggpubr* and *clusterProfiler* [45], respectively.

### Flux balance analysis

For our FBA simulations, we used the consensus genome-scale metabolic model of *Saccharomyces cerevisiae*, yeastGEM, version 8.3.0 [46]. The simulations were performed with the COBRApy python package [47], using yeastGEM definition of growth as the objective function to be maximized.

The upper bounds of reactions from yeastGEM were modified in correspondence with gene expression of related genes from our RNA-Seq data. For this integration of RNA-Seq and FBA we adapted the *E-Flux* method developed by *Colijn et al*. [29]. Briefly, every reaction is associated with a set of genes which products (enzymes or transporters) make the reaction possible. In the simplest case, only one gene or none at all are associated, meaning that the enzyme catalyzing the reaction is a single poly-peptide entity or that the reaction is spontaneous, respectively. When the enzymes are heteromeric the gene coding for the different subunits are associated by an “AND” keyword, and the maximum reaction flux was driven by the gene with the lowest expression of the set. When the reaction can be driven by more than one protein the corresponding gene (gene sets) are associated by the “OR” keyword, and the maximum reaction flux is a function of the sum of the corresponding gene (gene sets) expressions. If there was no expression value for a given gene the average expression of the corresponding experimental group was used instead.

The resulting upper reaction bounds were normalized between zero and 1000 (the default upper bound in the yeastGEM model). Two models came out as the result of this procedure, one for MCHM treated yeast and one for the untreated control.

Default solutions were determined for each model using the *optimize* method from COBRApy and with the default yeastGEM media. Phenotype phase plane of Growth vs D-Glucose exchange was calculated with the *production_envelope* method and the corresponding graphics generated with *ggpubr*.

Upper bounds of selected reactions were manually modified to test for the importance of such reactions in growth.

All fluxes are in mmol/(gDW*hour).

## Results

### MCHM affects yeast metabolism

To assess how MCHM treatment affects metabolism, 215 and 73 metabolites were identified by the ESI-MS and GC-MS procedures, respectively (S1 Table). The compounds from ESI-MS were dominated by phospholipids and sphingolipids, with 80 compounds belonging to those classes. In the GC-MS set, amino acids stand out, with 15 out of the 20 standard amino acids. There was almost no overlap between both sets of compounds, as only eight metabolites were detected by both procedures: adenosine, citric acid, L-lysine, L-proline, myristic, palmitic, and stearic acids and uridine. A total of 280 metabolites were consistently detected by our combined analysis (S1 Table), comprising a variety of lipid and polar compounds (S1 Table).

**Fig 1:**
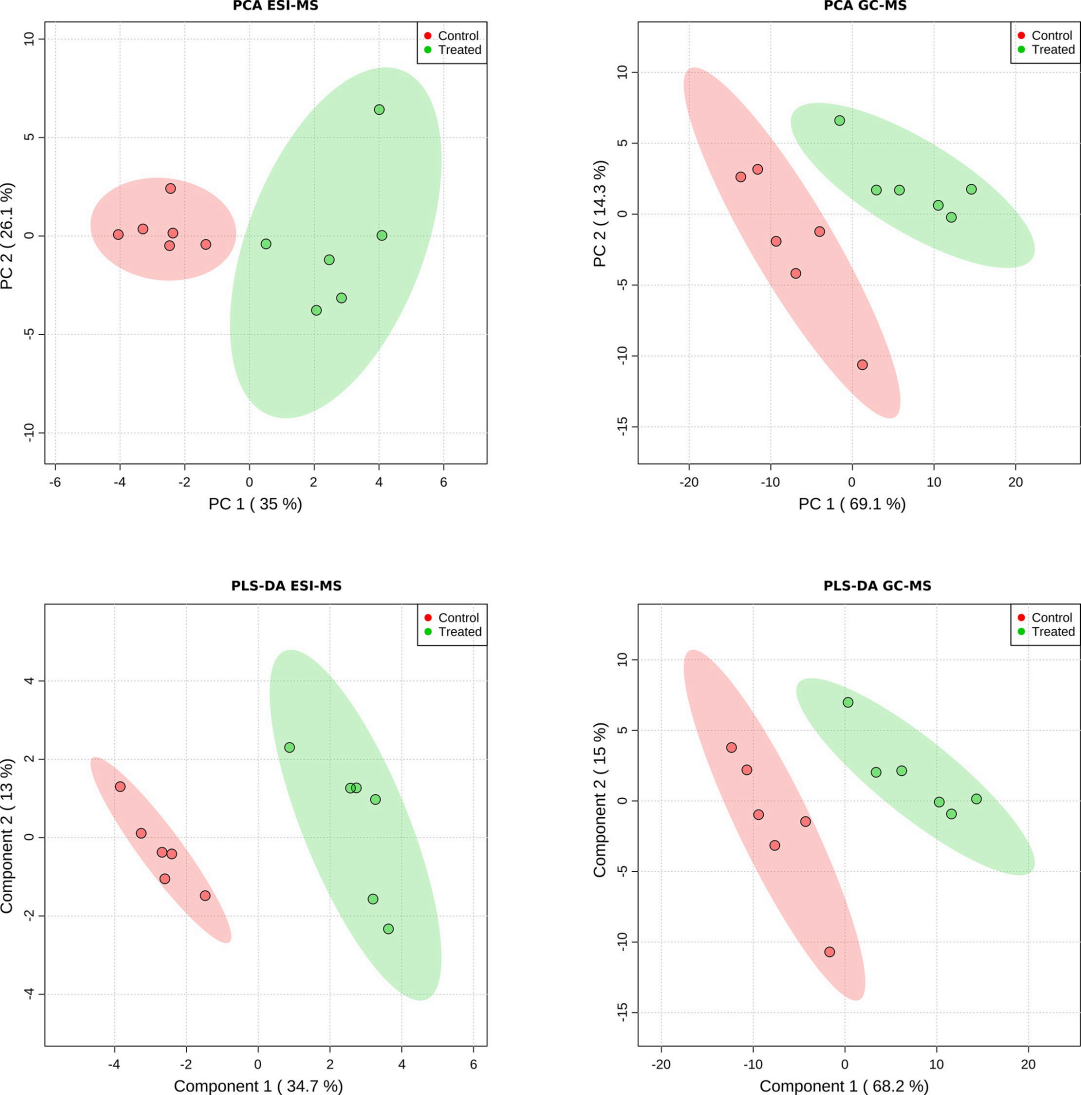
**Score plots from the Principal Component Analysis (PCA) (top) and the Partial Least Squares Discriminant Analysis (PLS-DA) (bottom), for ESI-MS (left) and GC-MS (right) data.** The 95% confidence areas are shown as well as the explained variance, shown in brackets in the corresponding axis labels.

Features from the MS spectra were detected, grouped, identified and their intensities calculated as described in Materials and Methods. Intensities were normalized to facilitate multivariate analysis (see Materials and Methods). Proper differentiation of the MCHM treated vs untreated control groups can be seen in the Principal Component Analysis (PCA, unsupervised, Fig 1 top) and the supervised methods Partial Least Squares Discriminant Analysis (PLS-DA, Fig 1 bottom), Orthogonal-Orthogonal Projections to Latent Structures Discriminant Analysis (OPLS-DA, S1 Fig, top) and Sparse Partial Least Squares—Discriminant Analysis (sPLS-DA, S1 Fig, bottom). The group separation (control vs treated) is consistent among the PCA and PCA-like analysis, which indicates that it reflects the effect of MCHM treatment and is independent of the supervision nature or the specificities of these PCA-like methodologies. The supervised methods validation can be seen in S2 Fig.

Relevant compounds were selected by a majority voting model, which takes into account the result of the t-test and eight multivariate analysis (PCA, PLS-DA, sPLS-DA, OPLS-DA, EBAM, Random Forest classification, SVM and SAM, see Materials and Methods, S2 and S3 Tables). For ESI-MS the number of significant compounds per analysis was: t-test (34, see S1 Table), PCA (30), PLS-DA (30), OPLS-DA (42), sPLS-DA (10), Random Forest (11, S3 Fig left), EBAM (33, S4 Fig left), SAM (39, S5 Fig left) and SVM (86). From these 26 metabolites were selected as relevant in the majority voting model (S2 Table). For GC-MS the numbers are: t-test (22, see S1 Table), PCA (23), PLS-DA (16), OPLS-DA (34), sPLS-DA (10), Random Forest (9, S3 Fig right), EBAM (23, S4 Fig right), SAM (29, S5 Fig right) and SVM (65). From these 23 metabolites were selected as relevant in the majority voting model (S3 Table).

**Fig 2:**
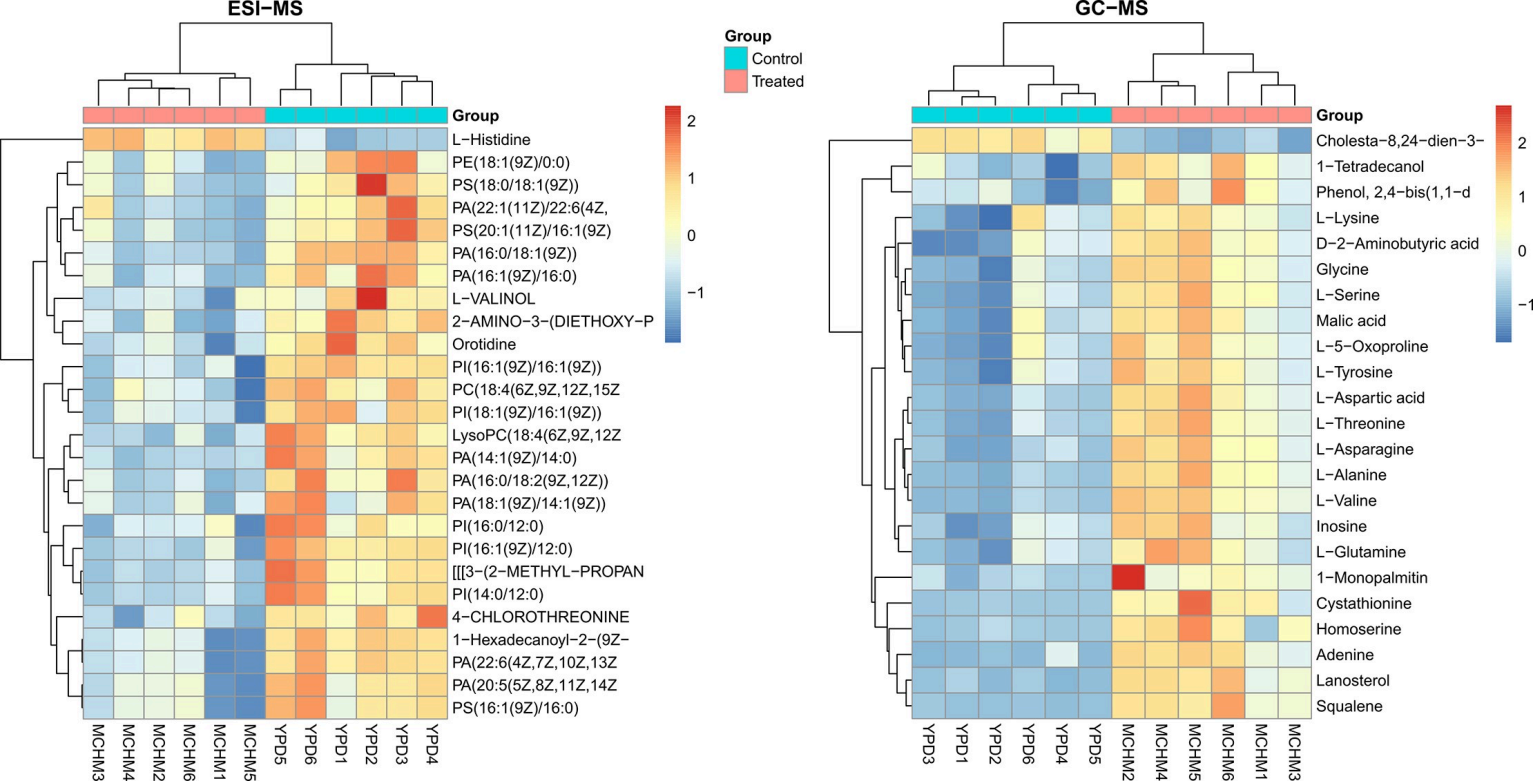
**Heatmap with the relevant compounds for ESI-MS (left) and GC-MS (right).** The cells are colored by the normalized intensities. Both the compounds (rows) and the samples (columns) are clustered and reordered by the similarity of the intensity patterns.

From the ESI-MS (left) and GC-MS (right) (Fig 2), 49 unique compounds were found relevant, with no common ones between ESI-MS and GC-MS. Samples were nicely clustered by groups in the heatmaps, in correspondence with what was previously observed in PCA-like analysis (Fig 1 and S1 Fig). The relevant compounds set from ESI-MS were dominated by glycerophospholipids (20 out of 26 compounds). The level of all these phospholipids was reduced due to MCHM treatment (Fig 2 left). Amino acids stood out in the GC-MS relevant set of metabolites, with 10 standards (A, D, T, V, N, G, Q, S, T and K) and two non-standard (5-Oxoproline or pyroglutamic acid and 2-aminobutyric acid, an alpha-amino acid derivative of alanine) which levels were increased due to the MCHM treatment (Fig 2 right). L-histidine, the only amino acid in the relevant set from ESI-MS and not detected by GC-MS, also has its levels increased due to MCHM treatment (Fig 2 left). Among the other metabolites which levels were also increased due to MCHM treatment are: homoserine (intermediate in the biosynthesis of methionine, threonine and isoleucine), cystathionine (an intermediate in the synthesis of cysteine), lanosterol (tetracyclic triterpenoid from which animal and fungal steroids are derived), squalene, adenine, inosine and malic acid (Fig 2 right and S3 Table). Besides the phospholipids, the nucleoside orotidine was among the metabolites with decreased level due to MCHM (Fig 2 left, S2 Table).

**Fig 3:**
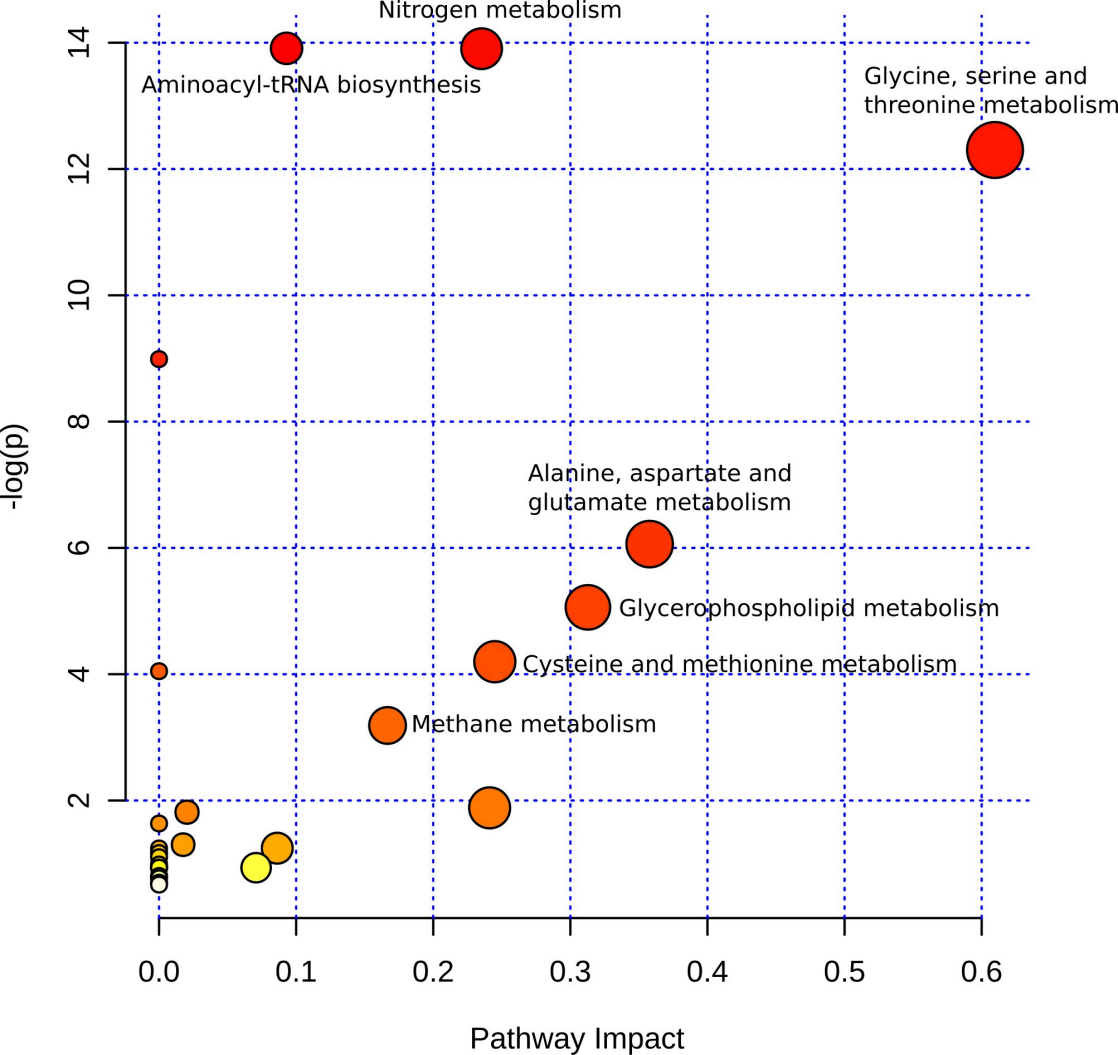
Pathway analysis using relevant metabolites from ESI-MS and GC-MS combined. The seven pathways with the impact greater than zero and p < 0.05 are labeled.

These relevant metabolites were used as input for pathway analysis (Fig 3), which combine pathway enrichment with pathway topology analysis. Seven metabolic pathways were both statistically significant and with impact (Fig 3). The relevant amino acids that dominated this analysis were from three pathways involved in the metabolism of amino acids: the aminoacyl t-RNA biosynthesis reactions have amino acids as reactants, the nitrogen metabolism has L-glutamine and 2-oxoglutarate as intermediaries, and L-serine, L-alanine, and glycine are involved in methane metabolism. The other relevant pathway was the glycerophospholipid metabolism, as expected due to number of glycerophospholipids affected by MCHM (Fig 2 left, S2 Table).

### Effect of MCHM on gene expression

We used a data set generated previously by our laboratory and available from https://www.ncbi.nlm.nih.gov/geo/query/acc.cgi?acc=GSE108873 [43]. For this analysis, we kept only the data regarding wild type S288c strain in YPD, treated or not with MCHM by 90 minutes.

**Fig 4:**
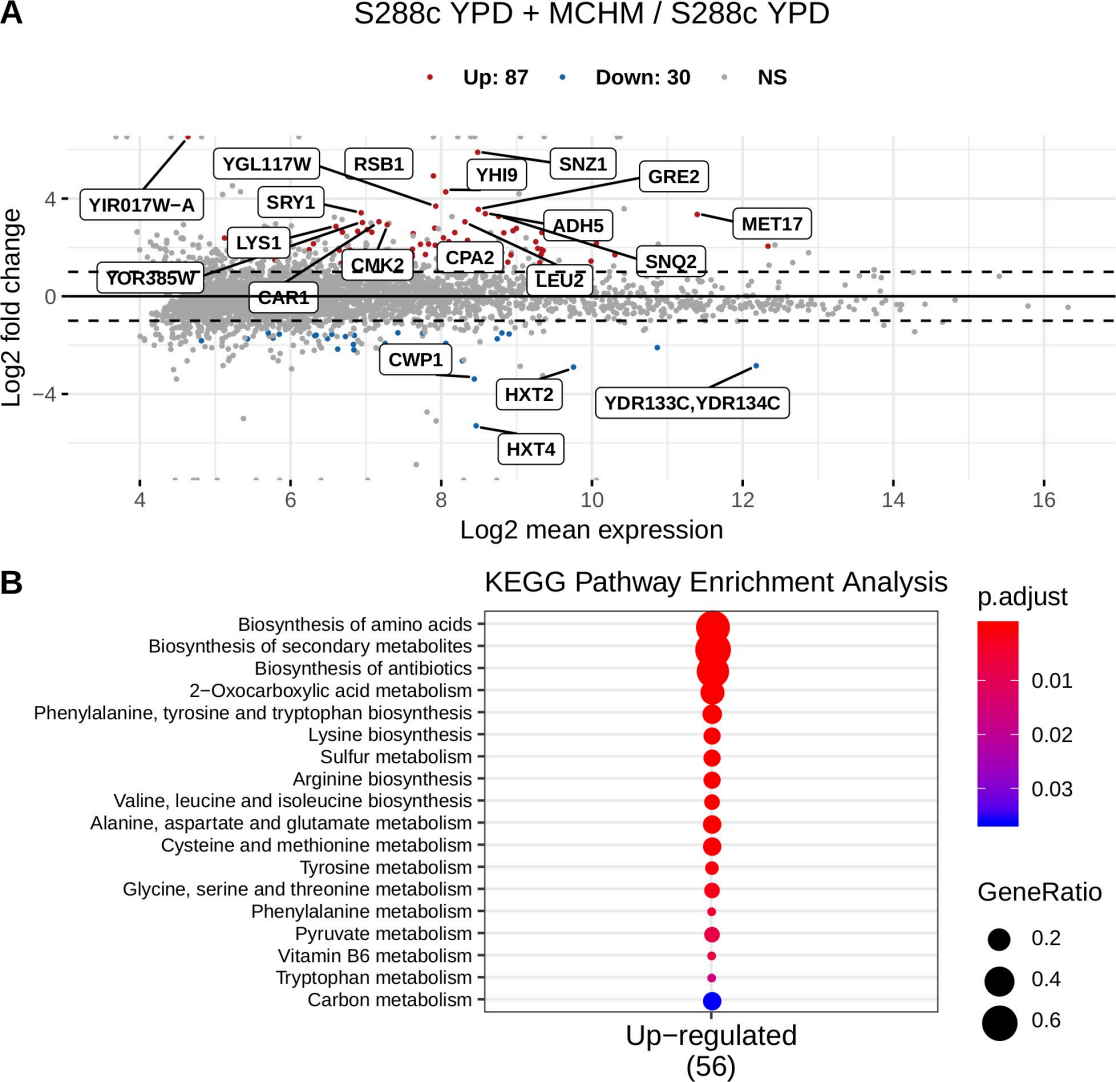
RNA-Seq gene expression data. MA plot (A). KEGG Pathway Enrichment Analysis for differentially expressed genes (B). No enrichment was found for downregulated genes.

From gene expression measurements for 3946 genes, 87 were upregulated and 30 downregulated due to MCHM treatment (Fig 4A, S4 Table) potentially affecting 18 metabolic pathways (Fig 4B), which include the three amino acid metabolism pathways in Fig 3. No pathway enrichment was found from the downregulated genes.

Seven downregulated genes were involved in ribosome biogenesis: *SDA1* and *RRP1*, involved in 60S ribosome biogenesis [48,49]. *ESF1*, its depletion causes severely decreased 18S rRNA levels [50]. *BFR2*, involved in pre-18S rRNA processing and component of SSU processome [51]. *MRD1*, required for the production of 18S rRNA and small ribosomal subunit [52]. *NOP4*, constituent of 66S pre-ribosomal particles and critical for large ribosomal subunit biogenesis and processing and maturation of 27S pre-rRNA [53]. *NOP7*, component of several pre-ribosomal particles [54]. Loss of *SDA1* function causes cells to arrest in G1 before Start and to remain uniformly as unbudded cells that do not increase significantly in size [55,56].

Among the rest of downregulated genes, there are two that encodes for cell wall mannoproteins (*CWP1* and *TIR1*) and three involved in iron and zinc transport and homeostasis (*FTR1*, *ZRT1*, and *IZH1*). MCHM affects the intracellular levels of iron and zinc [43].

The upregulated gene set was enriched in genes coding for enzymes of the amino acid biosynthesis pathways (28 out of 87) (Fig 4 B, S4 Table): *ARG1*, *ARG5*,*6*, *ARG7*, *CPA1*, *CPA2*, *ASN1*, *GDH1*, *HIS4*, *HIS5*, *HOM2*, *HOM3*, *LEU1*, *LEU2*, *LEU4*, *LYS1*, *LYS2*, *LYS12*, *MET5*, *MET6*, *MET17*, *MET22*, *TRP2*, *TRP5*, *TMT1*, *ARO1*, *ARO3*, *ADE3* and *THR4*. These gene products participate in the biosynthesis of the amino acids: D, R, N, E, H, M, T, L, K, C, W, Y, and F. Three other genes: *CAR1*, *MET3* and *MET14* are involved in R and M metabolism. *ARO8*, encoding for the aromatic aminotransferase I, was also upregulated and its expression is regulated by general control of amino acid biosynthesis [57].

Nine stress response-related genes are up-regulated due to MCHM treatment: *AHA1*, *GRE2*, *PDR3*, *PDR16*, *ICT1*, *TPO1*, *ENB1*, *SNQ2*, and *QDR3*.

It is of note that genes encoding for six mitochondrial enzymes (*MAE1*, *BAT1*, *ILV6*, *IDP1*, *GCV2*, and *LYS12*) and three mitochondrial transporters (*GGC1*, *OAC1*, and *ODC2*) were upregulated. From these, MAE1 codes for the mitochondrial malic enzyme which catalyzes the decarboxylation of malate to pyruvate (in addition to its key role in sugar metabolism, pyruvate is a precursor for synthesis of several amino acids); *BAT1* and *ILV6* products are involved in branched-chain amino acid biosynthesis and *ODC2* codes the 2-oxodicarboxylate transporter, which exports 2-oxoglutarate and 2-oxoadipate from the mitochondrial matrix to the cytosol for use in glutamate biosynthesis and in lysine metabolism.

### Modeling MCHM effect on yeast metabolism by flux balance analysis

Using the expression data and the gene rules from the yeastGEM model (version 8.3.0) upper bounds were calculated for 2504 reactions of the model. Two new metabolic models were created from the original yeastGEM model, named *control* and *treated*, with the upper bounds of their reaction fluxes calculated from the corresponding gene expression data (as explained in Materials and Methods), and using as the objective function the maximization of growth. A summary of the result of FBA simulations with these models is shown in Tables 1 and 2. All the input and output fluxes are shown, with the involved metabolites, the calculated flux rates, and their ranges. Flux ranges were calculated by Flux Variability Analysis with a fraction to the optimum of 1. The objective function flux is shown. The growth was predicted to decrease due to the MCHM treatment, from a flux of 0.0704 to 0.0591 mmol/(gDW*hour) (Tables 1 and 2). The flux ratio of growth between treated and control was ~0.839. So, MCHM treatment decreased yeast growth, consistent with the experimental results [43].

**Table 1:** FAB solution for the control model.

IN FLUXES			OUT FLUXES			OBJECTIVES	
Name	Flux	Range	Name	Flux	Range	Name	Flux
oxygen [e]	1.91	[1.91, 1.91]	H2O [e]	2.82	[2.23, 2.82]	growth	**0.0704**
phosphate [e]	1.2	[0.0178, 1.2]	formate [e]	1.74	[1.74, 1.74]		
D-glucose [e]	1	[1, 1]	carbon dioxide [e]	1.35	[1.35, 1.35]		
ammonium [e]	0.388	[0.388, 0.388]	diphosphate [e]	0.59	[0, 0.59]		
sulphate [e]	0.00538	0.00538, 0.00538]	H+ [e]	0.302	[0.302, 1.06]		
			ethanol [e]	0.17	[0.17, 0.17]		

[e] indicates extracellular compartment. All fluxes are in mmol/(gDW*hour).

**Table 2:** FAB solution for the treated model.

IN FLUXES			OUT FLUXES			OBJECTIVES	
Name	Flux	Range	Name	Flux	Range	Name	Flux
oxygen [e]	1.4	[1.4, 1.4]	H2O [e]	1.93	[1.77, 1.93]	growth	**0.0591**
D-glucose [e]	1	[1, 1]	carbon dioxide [e]	1.45	[1.45, 1.45]		
phosphate [e]	0.345	[0.015, 0.345]	formate [e]	1.25	[1.25, 1.25]		
ammonium[e]	0.325	[0.325, 0.325]	ethanol [e]	0.57	[0.57, 0.57]		
sulphate [e]	0.00451	[0.00451, 0.00451]	H+ [e]	0.212	[0.212, 1.38]		
			diphosphate [e]	0.165	[0, 0.165]		

[e] indicates extracellular compartment. All fluxes are in mmol/(gDW*hour).

**Fig 5:**
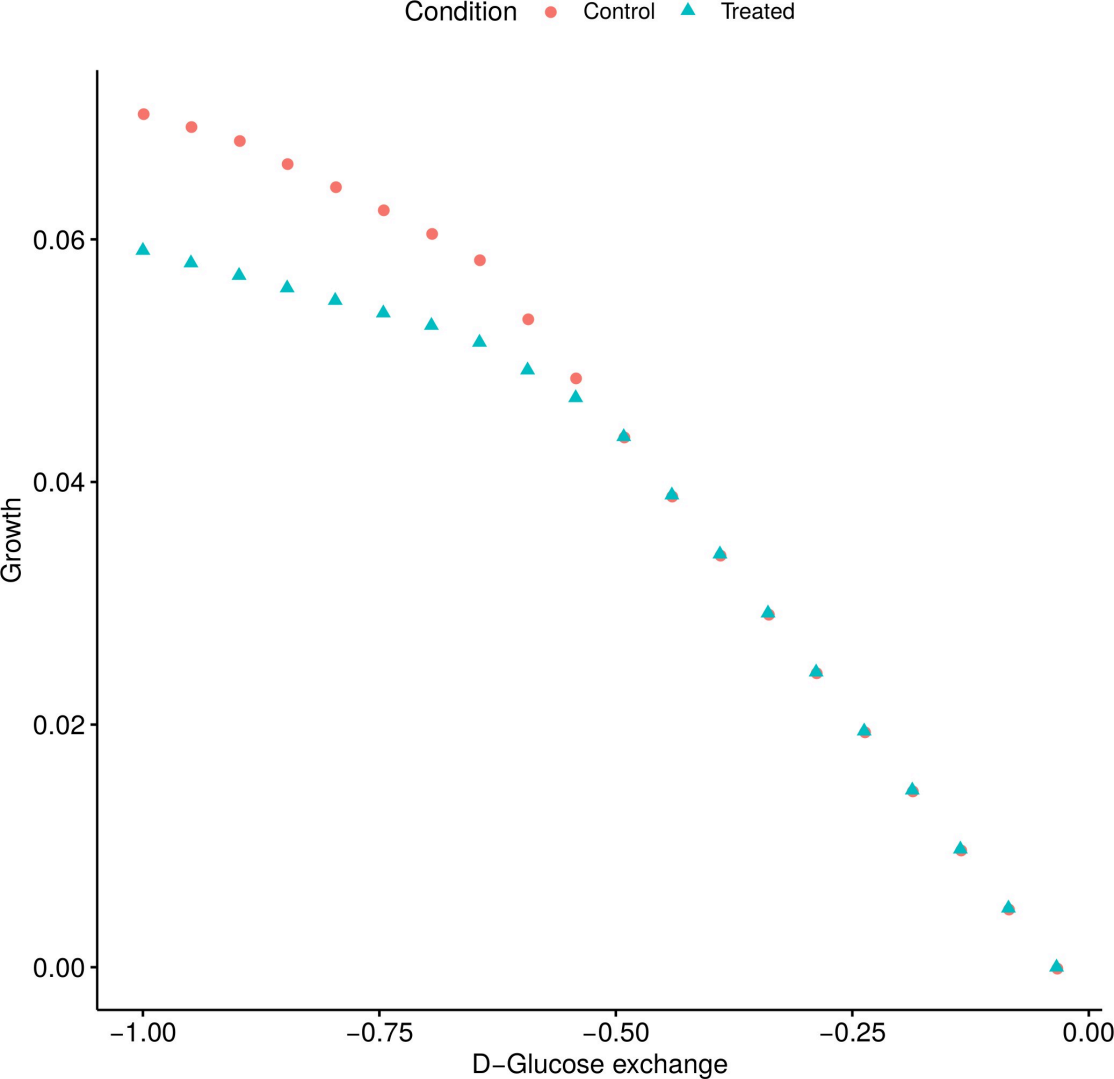
The effect of MCHM on yeast growth was predicted to depend on the concentration of D-Glucose in the medium. Phenotype phase plane of Growth vs D-Glucose exchange, from the FBA simulations with the control and treated models.

Our FBA simulations predict that the effect of MCHM on growth was diminished when the concentration of D-Glucose in the medium was decreased (Fig 5). There was a level of D-Glucose in the medium (~0.5 mmol/(gDW*hour)) from which the growth of the MCHM treated and control models were the same.

We then focused on the seven significant pathways from the pathway analysis (Fig 3), to analyze the flux ratios between the FBA solutions of the treated vs the control models. The Escher maps representations [42] of alanine, aspartate and glutamate metabolism, aminoacyl t-RNA biosynthesis, cysteine and methionine metabolism, glycerophospholipid metabolism, glycine, serine and threonine metabolism, methane metabolism and nitrogen metabolism are shown in S5–S12 Figs. As in any metabolic map, the nodes were the metabolites and the edges connecting them were the reactions, with arrowheads indicating the reaction direction and labeled by the corresponding enzyme or transporter. The ratios of the fluxes passing throughout the respective reactions in the MCHM treated vs untreated control models were shown next to the enzyme names, and the color and width of the edges were scaled in function of such ratio values. All the relevant pathways have fluxes affected due to the treatment, fluxes that involved some relevant metabolites from the metabolomics studies. Only two reactions in nitrogen metabolism pathway were relevant in the solutions of these FBA simulations: glutamine synthetase and bicarbonate formation reactions (S12 Fig). In the rest of the analyzed pathways most of the reactions were active (with non-zero net fluxes) (S6–S10 Figs). The fluxes of most reactions decreased in the MCHM treated model vs the control (flux ratios < 1). There were many reactions which flux ratio (treatment/control) was the same ratio of the growth, the value 0.839. The extreme case was aminoacyl t-RNA biosynthesis (S7 Fig), where all the reactions have this flux ratio. These reactions having the same treated/control flux ratio as the treated/control growth ratio indicated that they were linked to the growth but does not ensure that any of these reactions were actually limiting it.

#### Limiting reaction in FBA models

The reaction or reactions limiting the growth (limiting reactions) must be operating at the maximum allowed flux (upper bound value, calculated in function of the related gene expression levels) in the *treated* model. Two reactions operated at max flux in the model of the treatment (Table 3, last two rows). One of these, the ubiquinol:ferricytochrome c reductase, was also operating almost at maximum flux in the control model (Table 3, data row 3), and it was then the primary candidate to be the limiting reaction in our FBA simulations. Ubiquinol:ferricytochrome c reductase is part of the oxidative phosphorylation pathway and contributes to the proton gradient formation through the mitochondrial membrane.

**Table 3:** Reactions operating within 0.1 units of the maximum allowed flux.

Id	Name	Flux	Upper bound	Compartment	Model
r_0226	ATP synthase	4.375	4.375	m, c	Control
r_0438	ferrocytochrome-c:oxygen oxidoreductase	6.930	6.930	m, c	Control
r_0439	ubiquinol:ferricytochrome c reductase	3.465	3.536	m, c	Control
r_0501	glycine cleavage system	0.449	0.449	m	Control
r_0506	glycine-cleavage complex (lipoylprotein)	0.423	0.449	m	Control
r_0507	glycine-cleavage complex (lipoylprotein)	0.423	0.449	m	Control
r_0508	glycine-cleavage complex (lipoylprotein)	0.423	0.449	m	Control
r_0773	NADH:ubiquinone oxidoreductase	0.730	0.730	m	Control
r_1250	putrescine excretion	0.539	0.539	e, c	Control
r_0439	ubiquinol:ferricytochrome c reductase	2.582	2.582	m, c	Treated
r_0569	inorganic diphosphatase	0.330	0.330	m	Treated

Compartments legend: c, cytoplasm; e, extracellular; m, mitochondria. All fluxes are in mmol/(gDW*hour).

To test if *ubiquinol*:*ferricytochrome c reductase* was the limiting reaction we modified its upper bound in the control model to the one it has in the treated (Table 4, third data row vs first and second data row). The growth rate decreased from 0.0704 to 0.0597, which was practically the same growth of the treated model, 0.0591. As can be seen, modifying the maximum allowed flux of this reaction alone was enough to mimic the effect of the treatment in the growth, **confirming that ubiquinol:ferricytochrome c reductase was the limiting reaction** in our FBA simulations. We tried to recover the control phenotype (growth of 0.0704, Table 4, first data row) by setting the *ubiquinol*:*ferricytochrome c reductase* upper bound in the treated model to the one it had in the control one (Table 4, fourth data row vs first data row). The growth increased (up to 0.0672), but not at the level of the control model (not even after setting the upper bound to a higher value of 10, when the actual flux was lower than the set upper bound) (Table 4, data rows four and five). This means that in the treated model there were other reactions that become limiting when the maximum allowed flux through the ubiquinol:ferricytochrome c reductase was set higher. These reactions were the ATP synthase and the NADH:ubiquinone oxidoreductase, which were both operating at their maximum allowed flux in this condition (Table 5).

**Table 4:** Effect of ubiquinol:ferricytochrome c reductase reaction on growth.

Id	Name	Model	Upper bound	Actual flux	Growth
r_0439	ubiquinol:ferricytochrome c reductase	*Control*	*3*.*536*	*3*.*465*	*0*.*0704*
		*Treated*	*2*.*582*	*2*.*582*	*0*.*0591*
		Control	2.582	2.582	0.0597
		Treated	3.536	3.536	0.0672
		Treated	10.000	4.534	0.0688

The first two data rows show the upper bounds set for the reaction from the RNA-Seq data for the control and treated models, respectively, as well as the resulting actual fluxes and growth rates. The other three rows show the effect in the actual flux and on growth of modifying the upper bound values. All fluxes are in mmol/(gDW*hour).

**Table 5:** Potential limiting reactions in the treated model when the upper bound for the ubiquinol:ferricytochrome c reductase reaction was set to the one it has in the control model.

Id	Name	Flux	Upper bound	Compartment	Model
r_0226	ATP synthase	4.034	4.034	m, c	Treated
r_0439	ubiquinol:ferricytochrome c reductase	3.536	3.536	m, c	Treated
r_0773	NADH:ubiquinone oxidoreductase	0.918	0.918	m	Treated

All fluxes are in mmol/(gDW*hour).

Then, we kept the upper bound of ubiquinol:ferricytochrome c reductase reaction in the treated model set to 3.536 (the value from the control model, Table 4 data row one) and set the upper bounds of the other two reactions from Table 5 to an arbitrary large value (10), one at a time, to see if the control growth phenotype can be recovered (Table 6). Increasing the upper bounds of ubiquinol:ferricytochrome c reductase together with NADH:ubiquinone oxidoreductase increased to growth to 0.0672, which was still lower than the control growth rate (0.0704). But, increasing the upper bound of ubiquinol:ferricytochrome c reductase reaction together with the ATP synthase did recover the control growth phenotype, actually slightly improving the growth (0.0756 vs 0.0704) (Table 6).

**Table 6:** Recovering the growth phenotype in the treated model.

Id	Name	Model	Upper bound	Actual flux	Growth
r_0439	ubiquinol:ferricytochrome c reductase	Treated	3.536	3.536	0.0672
r_0773	NADH:ubiquinone oxidoreductase		10.000	1.445	
r_0439	ubiquinol:ferricytochrome c reductase	Treated	3.536	3.536	0.0756
r_0226	ATP synthase		10.000	4.863	

All fluxes are in mmol/(gDW*hour).

These results confirm than in our models the **ubiquinol:ferricytochrome c reductase was the limiting reaction**.

#### Limiting gene

The gene reaction rule for ubiquinol:ferricytochrome c reductase in the yeastGEM model is:

- (Q0105 and YBL045C and YDR529C and YEL024W and YEL039C and YFR033C and YGR183C and YHR001W-A and YJL166W and YOR065W and YPR191W) or (Q0105 and YBL045C and YDR529C and YEL024W and YFR033C and YGR183C and YHR001W-A and YJL166W and YJR048W and YOR065W and YPR191W)

This means that the protein responsible for carrying out the ubiquinol:ferricytochrome c reductase reaction is a multisubunit complex, with two possible quaternary structures, both conformed by polypeptides encoded by a set of 11 genes. The genes encoding for the components of the first quaternary structure were *COB* (Q0105), *COR1* (YBL045C), *QCR7* (YDR529C), *RIP1* (YEL024W), *CYC7* (YEL039C), *QCR6* (YFR033C), *QCR9* (YGR183C), *QCR10* (YHR001W-A), *QCR8* (YJL166W), *CYT1* (YOR065W) and *QCR2* (YPR191W). The genes encoding for the second were *COB* (Q0105), *COR1* (YBL045C), *QCR7* (YDR529C), *RIP1* (YEL024W), *QCR6* (YFR033C), *QCR9* (YGR183C), *QCR10* (YHR001W-A), *QCR8* (YJL166W), *CYC1* (YJR048W), *CYT1* (YOR065W) and *QCR2* (YPR191W). The maximum flux of a multisubunit complex will depend on the gene with the lowest average expression, which will be the limiting factor of the complex assembling. For both possible complex configurations, in both control and treatment conditions, ***CYT1* (YOR065W)** had the lowest average expression (Table 7). The expression level of *CYT1* was limiting the maximum flux allowed through the ubiquinol:ferricytochrome c reductase reaction in our FBA simulations. We were able to reproduce the results shown in Tables 4–6, by modifying *CYT1* expression values used to build the control and treated models, instead of the derived reaction upper bound.

**Table 7:** Gene average expression for components of the ubiquinol:Ferricytochrome c reductase complex.

Gene id	Gene	Expression control	Expression treated	Complex configuration
YBL045C	COR1	134.50	122.91	1 and 2
*YOR065W*	*CYT1*	*122*.*69*	*89*.*59*	*1 and 2*
YPR191W	QCR2	133.78	138.20	1 and 2
YFR033C	QCR6	125.40	107.32	1 and 2
YJL166W	QCR8	317.91	552.80	1 and 2
YEL024W	RIP1	160.27	188.18	1
YJR048W	CYC1	309.61	179.50	2
YDR529C	QCR7	407.45	555.42	2
Average	All genes without expression data	472.26	448.02	1 and 2

The enzyme has two possible quaternary structures, labeled as complex configuration 1 and 2 in this table. The presence of the genes in a given configuration is stated in the last column.

## Discussion

MCHM significantly affected amino acid metabolism, increasing the total intracellular concentration of 11 out of 20 standard amino acids. As 28 genes coding for enzymes of the amino acid biosynthesis pathways were upregulated due to MCHM treatment, the higher levels of such amino acids can be partially explained by their probable increased biosynthesis. The other contributing factor could be a reduced protein production, due to the deleterious effect of MCHM on ribosome biogenesis (downregulating seven critical genes of the process), leading to amino acid accumulation. The downregulation of ribosome biogenesis is the first step in stress response such as starvation, heat, or chemical. To respond to stress, energy-intensive functions are down-regulated and inhibition of rRNA occurs in less than ten minutes of dextrose depletion [58].

There is evidence of other cellular stressors which also variate the levels of some amino acids. Cu^2+^ increased the levels of L-glutamate, L-phenylalanine, and L-leucine and decreased the level of L-aspartate in *S*. *cerevisiae* [18]. From these only L-aspartate varied in our analysis increased its levels due to MCHM.

*MCHM treatment provokes the upregulation of nine genes related to the stress response*. From these genes, *AHA1* encodes a co-chaperone that binds Hsp82 and its expression is regulated by stresses such as heat shock [59]. *GRE2* encodes the 3-methylbutanal reductase and its expression is induced by oxidative, ionic, osmotic, heat shock and heavy metals stress [60]. *PDR3* is a transcriptional activator of the pleiotropic drug resistance network [61]. *PDR16* encodes the phosphatidylinositol transfer protein and it is controlled by the multiple drug resistance regulator Pdr1p. It affects lipid biosynthesis and resistance to multiple drugs [61]. *SNQ2* and *QDR3* encode multidrug transporters involved in multidrug resistance [62,63]; *ENB1* encodes for an endosomal ferric enterobactin transporter, which is expressed under conditions of iron deprivation [64]; *TPO1* codes for a polyamine transporter which exports spermine and spermidine from the cell during oxidative stress, controlling the timing of expression of stress-responsive genes [65]; *ICT1* codes the lysophosphatidic acid acyltransferase responsible for enhanced phospholipid synthesis during organic solvent stress [66]⁠.

We did not detect enhanced phospholipid biosynthesis in our metabolomics analysis, by the contrary, the levels of all glycerophospholipids included in the relevant metabolites were decreased due to MCHM, while the levels of the remaining phospholipids did not change. The reduced levels of these molecules of phosphatidylethanolamine, phosphatidylinositol, and phosphatidylserine in MCHM treated cells point toward a significant effect of MCHM in yeast cellular membranes, with potential effects on their biophysical properties, which could impact several cellular processes involving membranes. *In vitro* MCHM acts as a hydrotrope, a compound that increases the solubility of proteins by inducing liquid-liquid phase transitions [43]. At high protein concentrations proteins can aggregate which is generally thought to inactive enzymatic activities (reviewed in [67]). The wide range of pathways affected by MCHM could be contributed to its nonspecific ability to alter protein structure.

The FBA simulations done with genome-scale metabolic network models (GSMNM) of MCHM treated vs non-treated control yeast were able to reproduce the deleterious effect of MCHM on cell’s growth. These GSMNM integrated the gene expressions from the RNA-Seq data, as explained in Materials and Methods. The flux ratio through several reactions in the six significant pathways from the metabolomics analysis was linked to the simulated growth ratio in MCHM-treated vs untreated control models, but this does not indicate causality. The FBA simulations suggest a critical role to the ubiquinol:ferricytochrome c reductase as the enzyme catalyzing the limiting reaction which determined the reduced growth in MCHM. From this multisubunit complex *CYT1* product was the component limiting the overall reaction flow, and the lower expression of *CYT1* due to MCHM can explain the lower growth, at least in the FBA simulations. It is of note that the fold change of the expression levels of *CYT1* was not large enough (logFC < 2) for the gene to reach the cutoff as relevant from the RNA-Seq data, but the GSMNM created were very sensitive to its levels. This highlight the extra value of RNA-Seq data integration in FBA simulations, allowing to assess the impact of gene levels in whole-cell functional environment, where apparently irrelevant genes can prove to be the driven force behind observed phenotypes. Transcription of *CYT1* is positively controlled by oxygen in the presence of glucose, through the haem signal and mediated by the transcription factor, Hap1 [68]. It is additionally regulated by the HAP2/3/4 complex which mediates gene activation mainly under glucose-free conditions. *CYT1* basal transcription is partially affected by Cpf1, transcription factor required for regulation of methionine biosynthetic genes [68].

The other significant reaction that came from the FBA analysis was the ATP synthase, which maximum allowed flux or upper bound was required to be increased together with the one of ubiquinol:ferricytochrome c reductase to rescue the control growth phenotype in the MCHM treated model. Combining flux balance analysis with *in vitro* measured enzyme specific activities it was determined that fermentation was more catalytically efficient than respiration [69], producing more ATP per mass of required enzymes. In that study the enzyme F1F0-ATP synthase was found to have flux control over respiration in the model, causing the Crabtree Effect [69].

## Conclusions

MCHM produced amino acid accumulation in *S*. *cerevisiae*, affecting several amino acid-related metabolic pathways and probably slowing down protein biosynthesis due to the downregulation of genes related to ribosome biogenesis. MCHM affects phospholipid biosynthesis, reducing the levels of different molecules of phosphatidylethanolamine, phosphatidylinositol, and phosphatidylserine, which should affect cellular membranes composition and their biophysical properties. The FBA simulations suggest that the lower flow through ubiquinol:ferricytochrome c reductase reaction, caused by the MCHM-provoked under-expression of *CYT1* gene, could be the driven force behind the observed effect on yeast metabolism and growth.

## Supporting information

S1 Fig **Score plots from the Orthogonal-Orthogonal Projections to Latent Structures Discriminant Analysis (OPLS-DA) (top) and the Sparse Partial Least Squares—Discriminant Analysis (sPLS-DA) (bottom), for ESI-MS (left) and GC-MS (right) data.** The 95% confidence areas are shown as well as the explained variance, shown in brackets in the corresponding axis labels.
(TIF)

S2 Fig Supervised models validation. PLS-DA models validation by permutation tests based on separation distance for ESI-MS (A) and GC-MS (B). OPLS-DA models validation by permutation tests, showing the observed and cross-validated R2Y and Q2 coefficients, for ESI-MS (C) and GC-MS (E). Plot of the performance of the sPLS-DA models evaluated using leave-one-out cross-validations with increasing numbers of components, for ESI-MS (E) and GC-MS (F).
(TIF)

S3 Fig **Significant features identified by Random Forest for A) ESI-MS and B) GC-MS data.** The features are ranked by the mean decrease in classification accuracy when they are permuted.
(TIF)

S4 Fig **Empirical Bayesian Analysis of Microarray (EBAM) for A) ESI-MS and B) GC-MS data.** 33 and 23 significant compounds are identified with this method for ESI-MS and GC-MS, respectively.
(TIF)

S5 Fig **Significance Analysis of Microarray (SAM) for A) ESI-MS and B) GC-MS data.** The green circles represent features that exceed the specified threshold. 39 and 29 significant features are identified by SAM from ESI-MS and GC-MS respectively.
(TIF)

S6 Fig Escher map of the alanine, aspartate, and glutamate metabolism. The flux ratios between treated and control model FBA solutions are represented. Edges' thickness and color are a function of the respective ratio values. The ratio value of 0.839 is common among the map.
(TIF)

S7 Fig Escher map of the aminoacyl t-RNA biosynthesis. The flux ratios between treated and control model FBA solutions are represented. Edges' thickness and color are a function of the respective ratio values. All the reactions have a ratio value of 0.839.
(TIF)

S8 Fig Escher map of the cysteine and methionine metabolism. The flux ratios between treated and control model FBA solutions are represented. Edges' thickness and color are a function of the respective ratio values. The ratio value of 0.839 is common among the map.
(TIF)

S9 Fig Escher map of the glycerophospholipid metabolism. The flux ratios between treated and control model FBA solutions are represented. Edges' thickness and color are a function of the respective ratio values. The glycerol-3-phosphate dehydrogenase reactions has a ratio value of 0.839.
(TIF)

S10 Fig Escher map of the glycine, serine and threonine metabolism. The flux ratios between treated and control model FBA solutions are represented. Edges' thickness and color are a function of the respective ratio values. The ratio value of 0.839 is common among the map.
(TIF)

S11 Fig Escher map of the methane metabolism. The flux ratios between treated and control model FBA solutions are represented. Edges' thickness and color are a function of the respective ratio values. Only a fraction of KEGG’s reference pathway is present in yeast.
(TIF)

S12 Fig Escher map of the nitrogen metabolism. The flux ratios between treated and control model FBA solutions are represented. Edges' thickness and color are a function of the respective ratio values.
(TIF)

S1 Table List of all metabolites detected by ESI-MS and GC-MS. The experiment of origin is indicated as well as the following values from the comparison of their levels in the MCHM treated vs control samples: p-value (t-test), adjusted p values (“BH”), q-values, log2 of the fold change, and the coefficient of variation of the controls.
(XLSX)

S2 Table Majority voting model for relevant compounds selection from ESI-MS data. The 128 compounds being labeled as significant for at least one of the nine analyses used are shown. For each analysis is indicated if the respective compound is significant (1) or not (0). These values are added for the final votes. Compound with a majority of votes (5 or more) are selected as relevant (highlighted in yellow, “Selected” column value equals TRUE), for a total of 26 compounds.
(XLSX)

S3 Table Majority voting model for relevant compounds selection from GC-MS data. The 66 compounds being labeled as significant for at least one of the nine analyses used are shown. For each analysis is indicated if the respective compound is significant (1) or not (0). These values are added for the final votes. Compound with the majority of votes (5 or more) are selected as relevant (highlighted in yellow, “Selected” column value equals TRUE), for a total of 23 compounds.
(XLSX)

S4 Table Genes up and down-regulated due to MCHM treatment. The fold change and adjusted p values are provided, as well as a functional annotation, when available.
(XLSX)
