# Supplementary figures and images for: Effects of MCHM on yeast metabolism
Source: PLoS One. 2019 Oct 17;14(10):e0223909. doi: 10.1371/journal.pone.0223909 (PMC6797124; doi:10.1371/journal.pone.0223909)

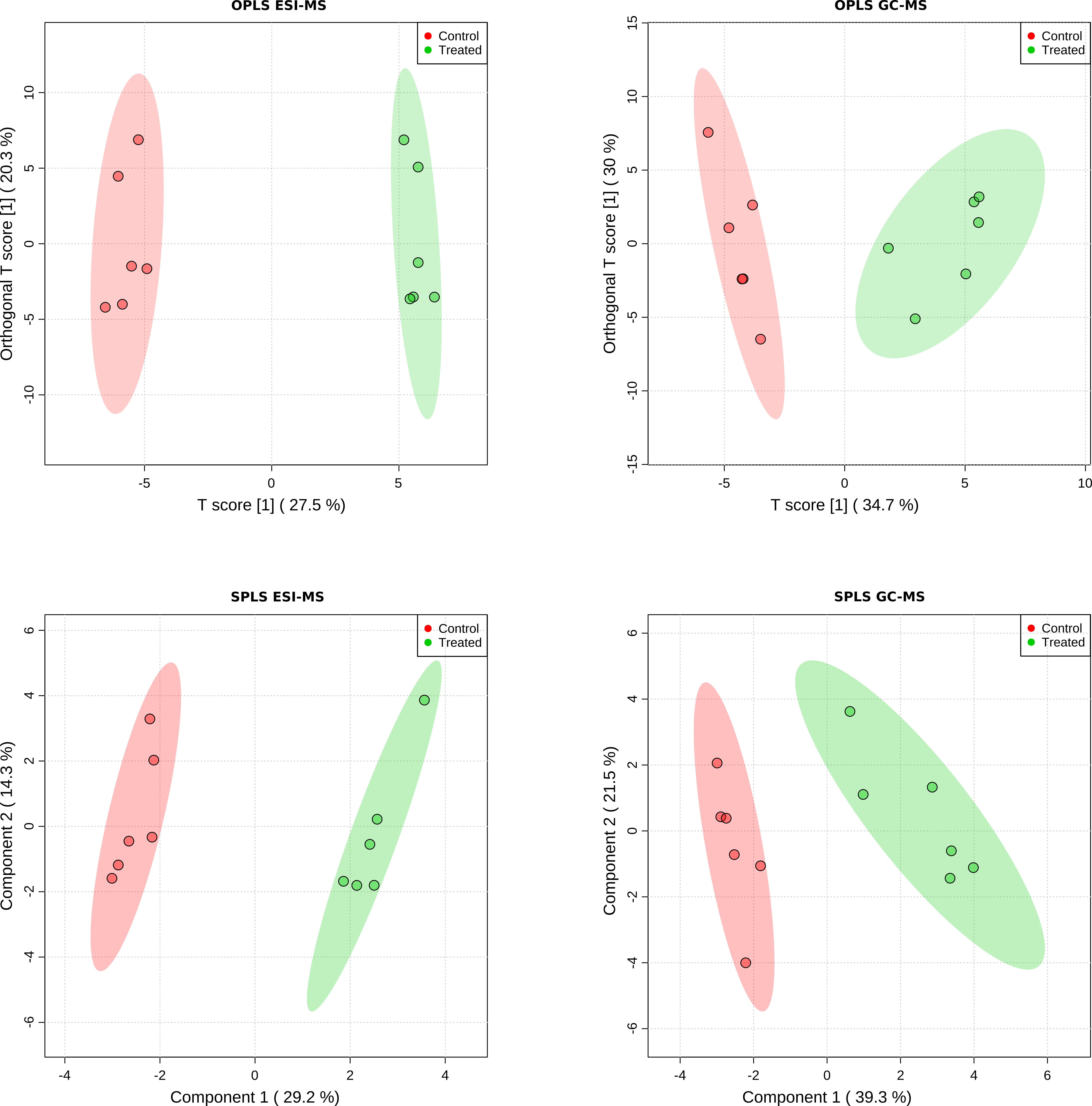

Supplement: S1 Fig — Score plots from the Orthogonal-Orthogonal Projections to Latent Structures Discriminant Analysis (OPLS-DA) (top) and the Sparse Partial Least Squares—Discriminant Analysis (sPLS-DA) (bottom), for ESI-MS (left) and GC-MS (right) data. The 95% confidence areas are shown as well as the explained variance, shown in brackets in the corresponding axis labels. (TIF) [file pone.0223909.s001.tif]

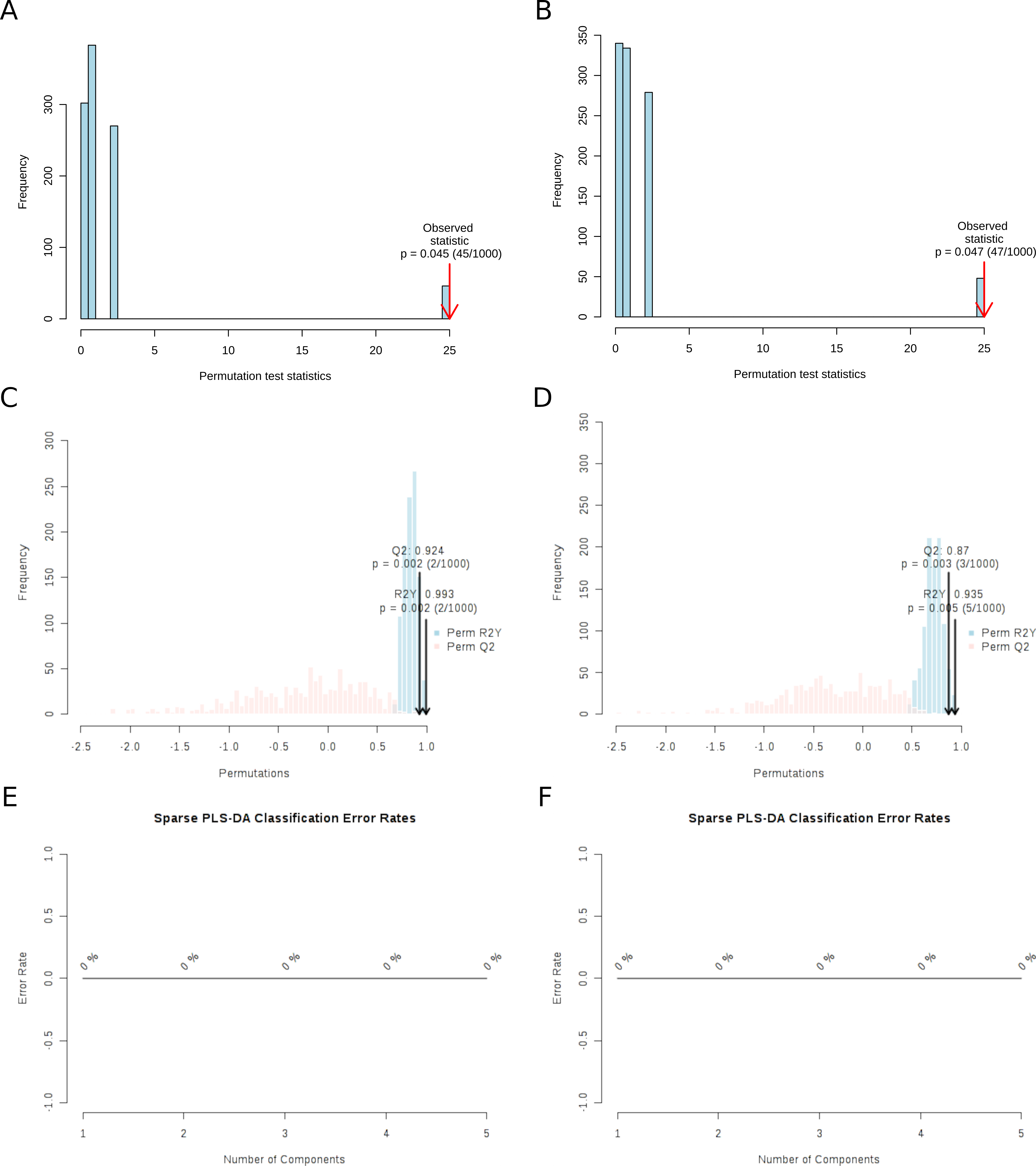

Supplement: S2 Fig — PLS-DA models validation by permutation tests based on separation distance for ESI-MS (A) and GC-MS (B). OPLS-DA models validation by permutation tests, showing the observed and cross-validated R2Y and Q2 coefficients, for ESI-MS (C) and GC-MS (E). Plot of the performance of the sPLS-DA models evaluated using leave-one-out cross-validations with increasing numbers of components, for ESI-MS (E) and GC-MS (F). (TIF) [file pone.0223909.s002.tif]

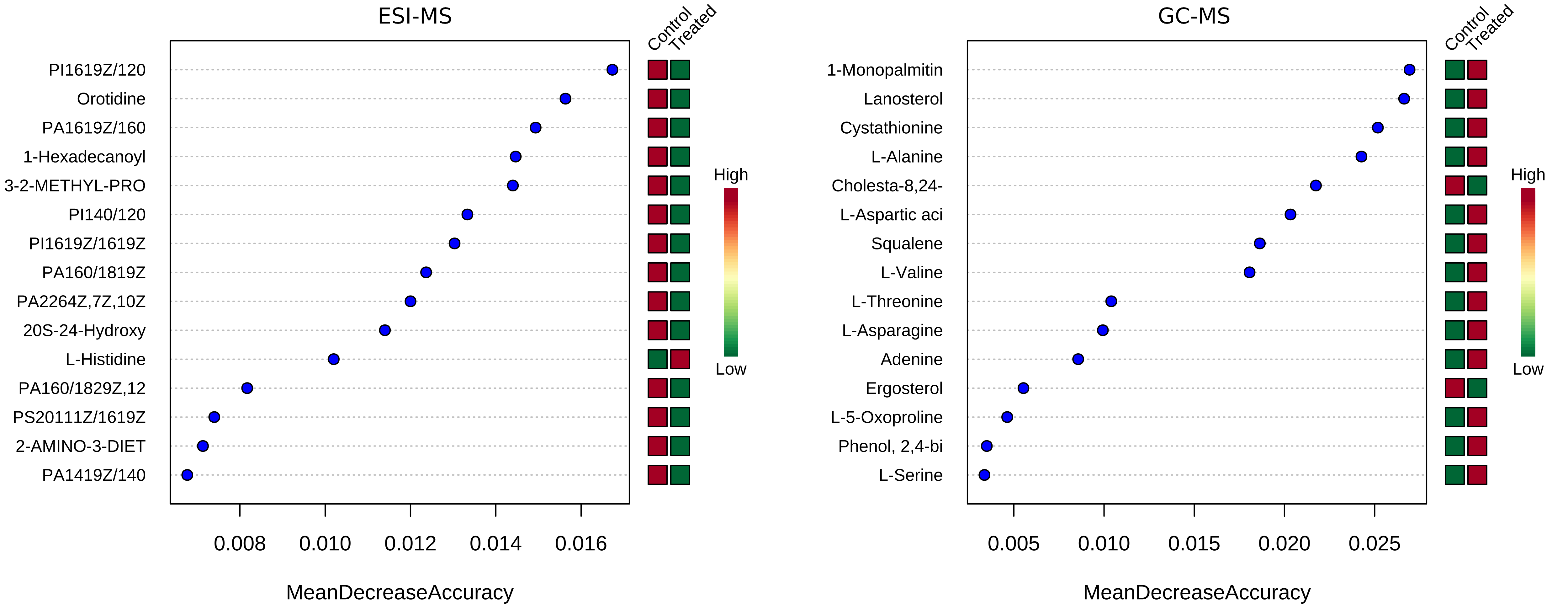

Supplement: S3 Fig — Significant features identified by Random Forest for A) ESI-MS and B) GC-MS data. The features are ranked by the mean decrease in classification accuracy when they are permuted. (TIF) [file pone.0223909.s003.tif]

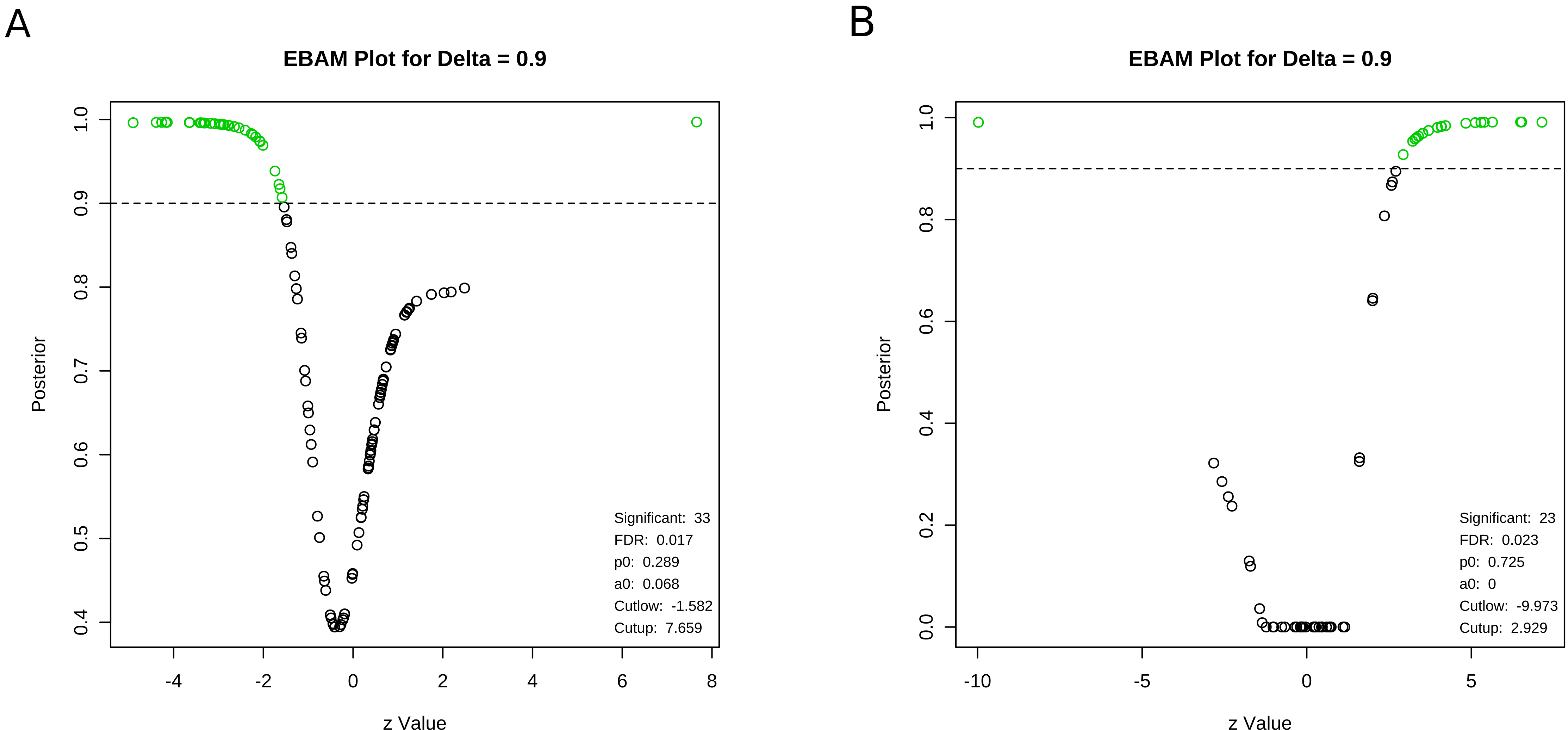

Supplement: S4 Fig — Empirical Bayesian Analysis of Microarray (EBAM) for A) ESI-MS and B) GC-MS data. 33 and 23 significant compounds are identified with this method for ESI-MS and GC-MS, respectively. (TIF) [file pone.0223909.s004.tif]

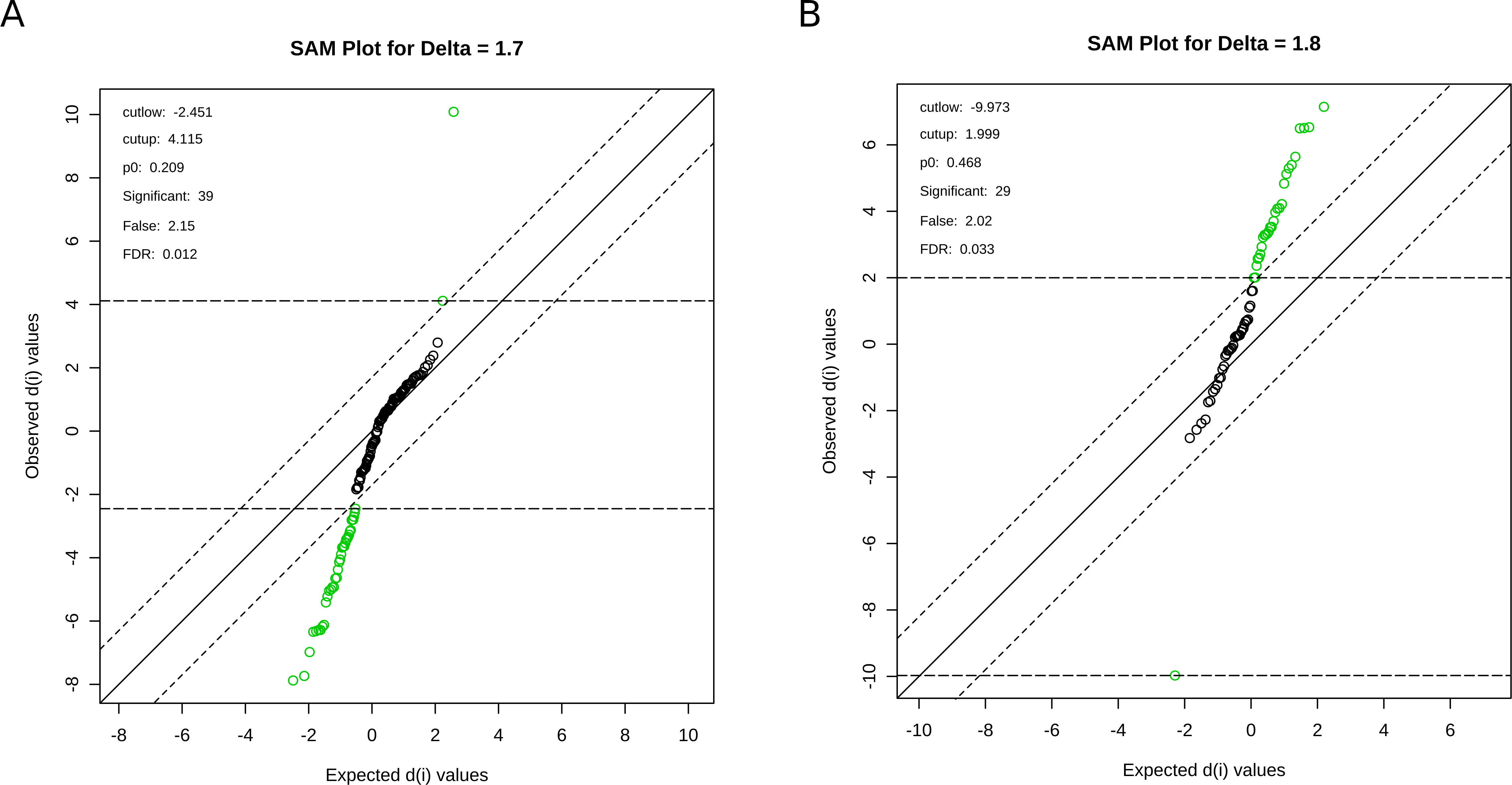

Supplement: S5 Fig — Significance Analysis of Microarray (SAM) for A) ESI-MS and B) GC-MS data. The green circles represent features that exceed the specified threshold. 39 and 29 significant features are identified by SAM from ESI-MS and GC-MS respectively. (TIF) [file pone.0223909.s005.tif]

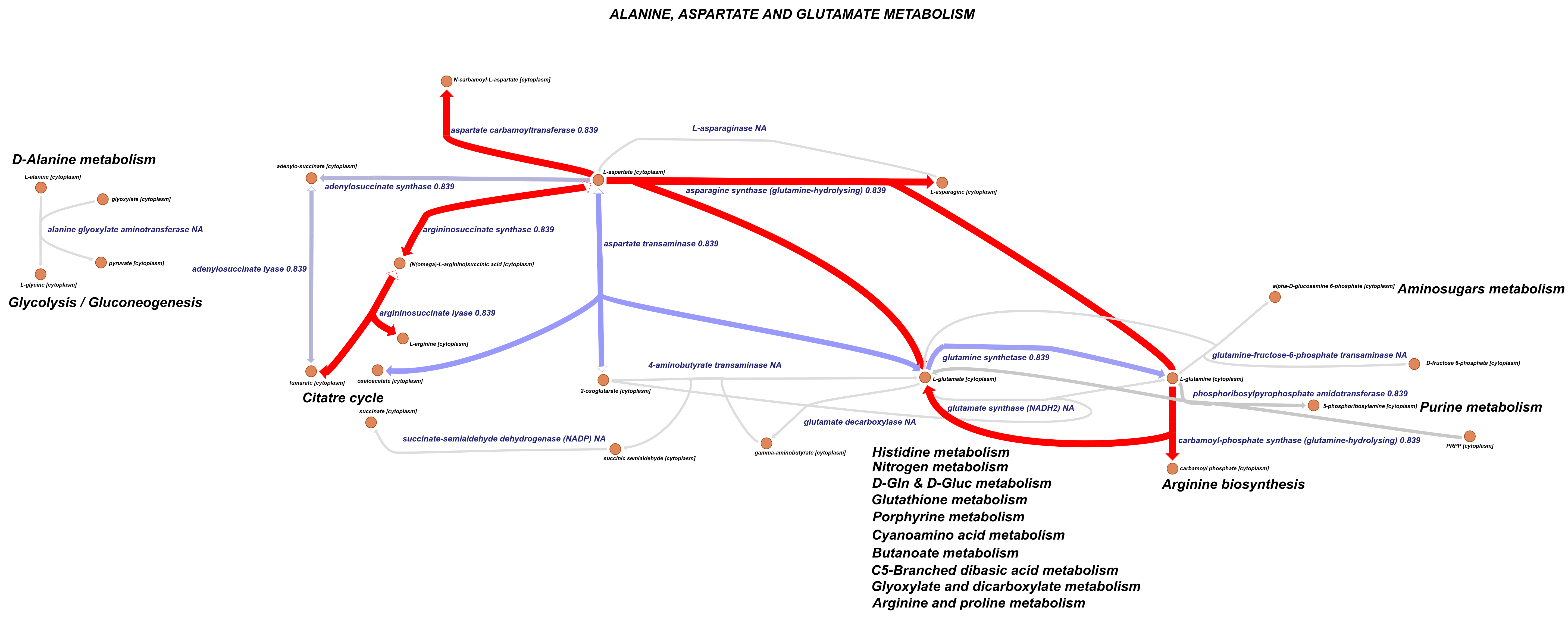

Supplement: S6 Fig — The flux ratios between treated and control model FBA solutions are represented. Edges' thickness and color are a function of the respective ratio values. The ratio value of 0.839 is common among the map. (TIF) [file pone.0223909.s006.tif]

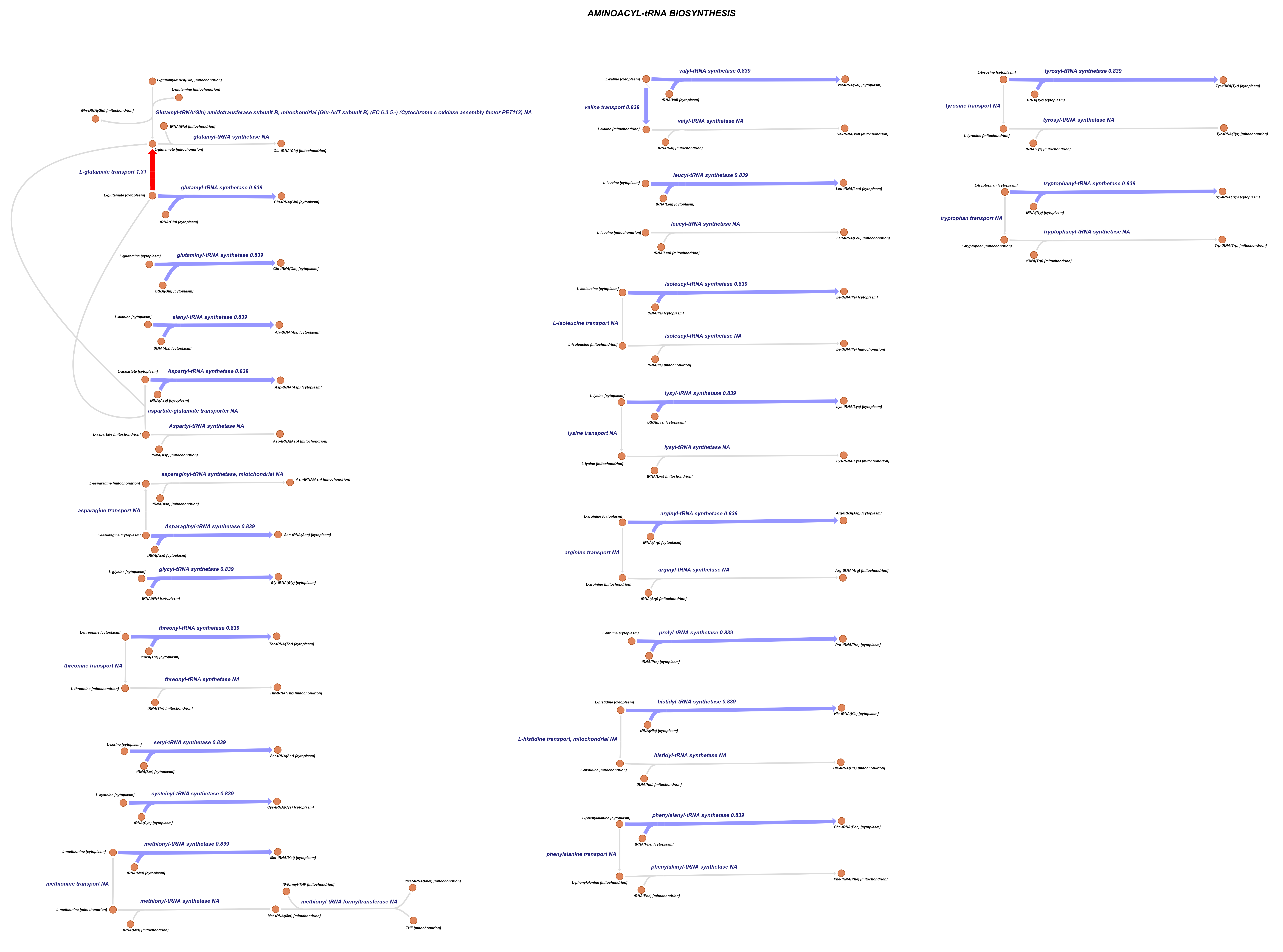

Supplement: S7 Fig — The flux ratios between treated and control model FBA solutions are represented. Edges' thickness and color are a function of the respective ratio values. All the reactions have a ratio value of 0.839. (TIF) [file pone.0223909.s007.tif]

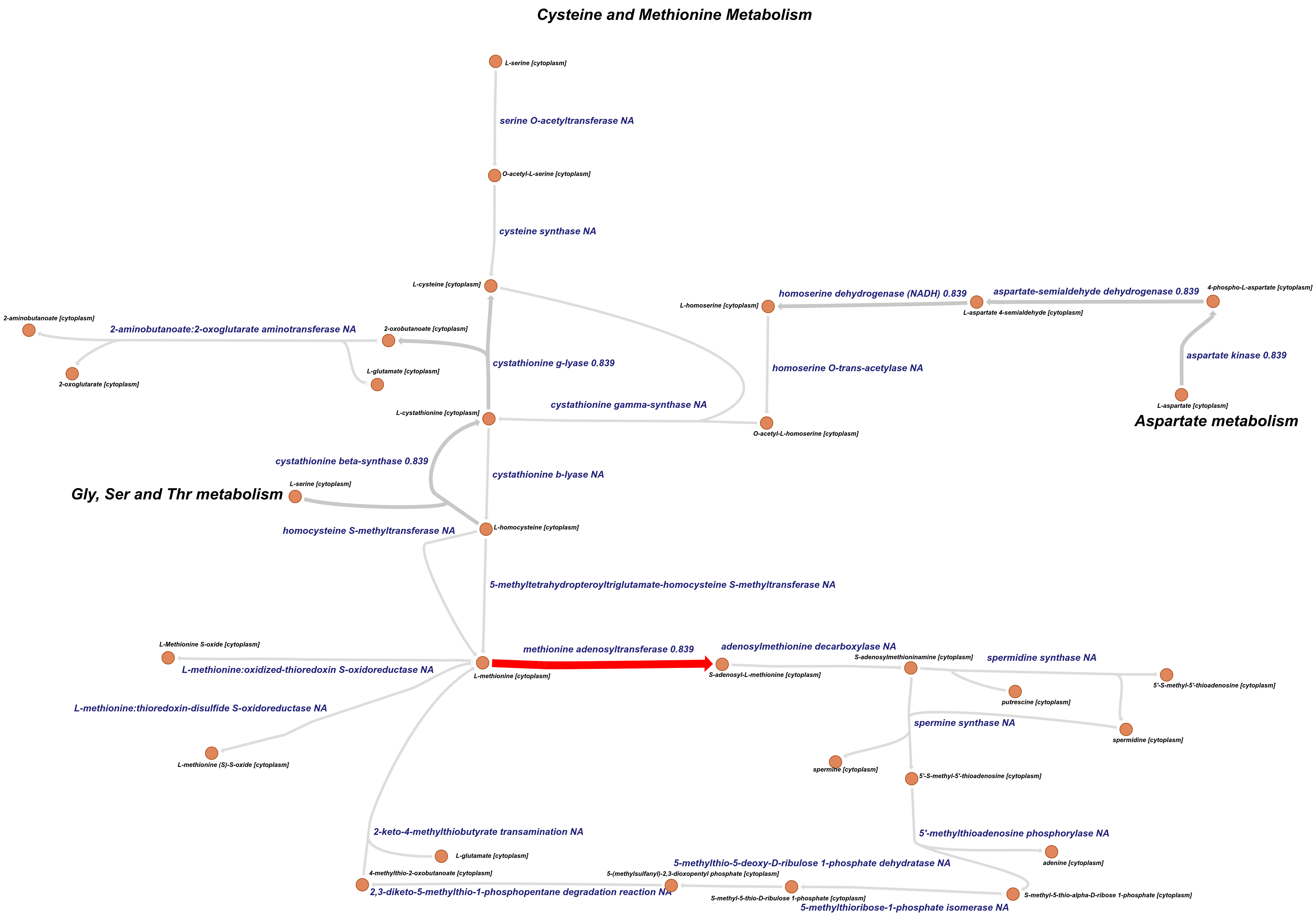

Supplement: S8 Fig — The flux ratios between treated and control model FBA solutions are represented. Edges' thickness and color are a function of the respective ratio values. The ratio value of 0.839 is common among the map. (TIF) [file pone.0223909.s008.tif]

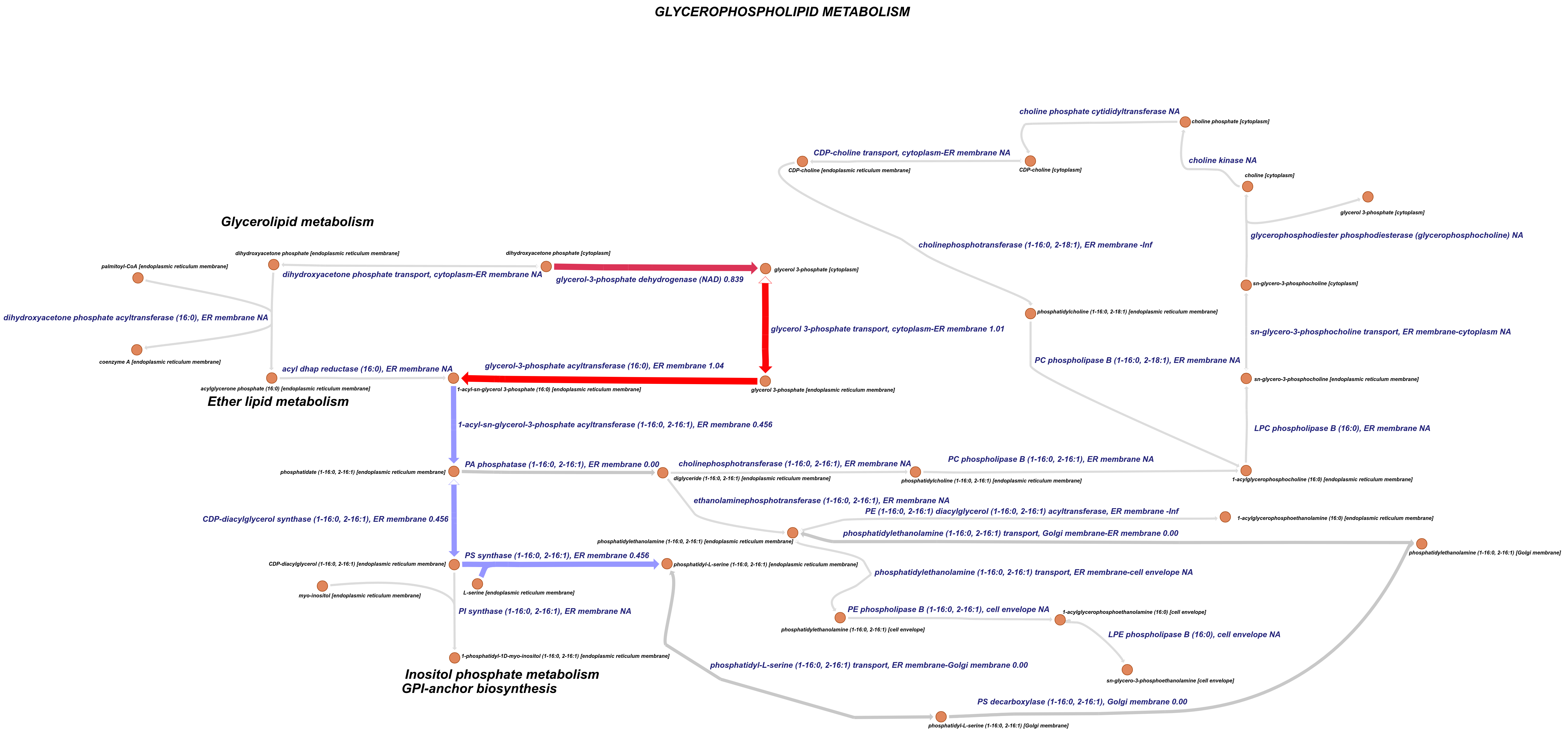

Supplement: S9 Fig — The flux ratios between treated and control model FBA solutions are represented. Edges' thickness and color are a function of the respective ratio values. The glycerol-3-phosphate dehydrogenase reactions has a ratio value of 0.839. (TIF) [file pone.0223909.s009.tif]

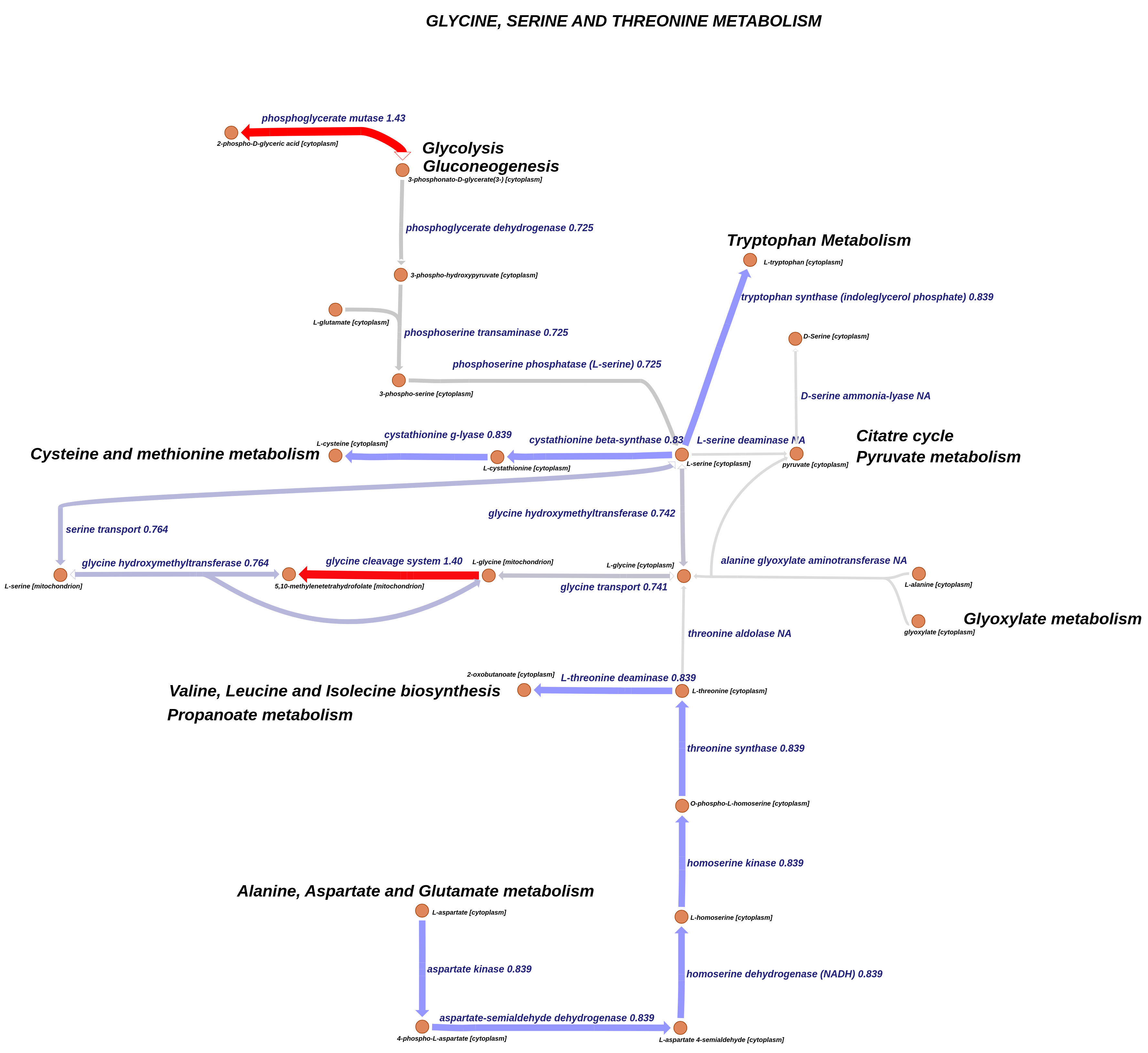

Supplement: S10 Fig — The flux ratios between treated and control model FBA solutions are represented. Edges' thickness and color are a function of the respective ratio values. The ratio value of 0.839 is common among the map. (TIF) [file pone.0223909.s010.tif]

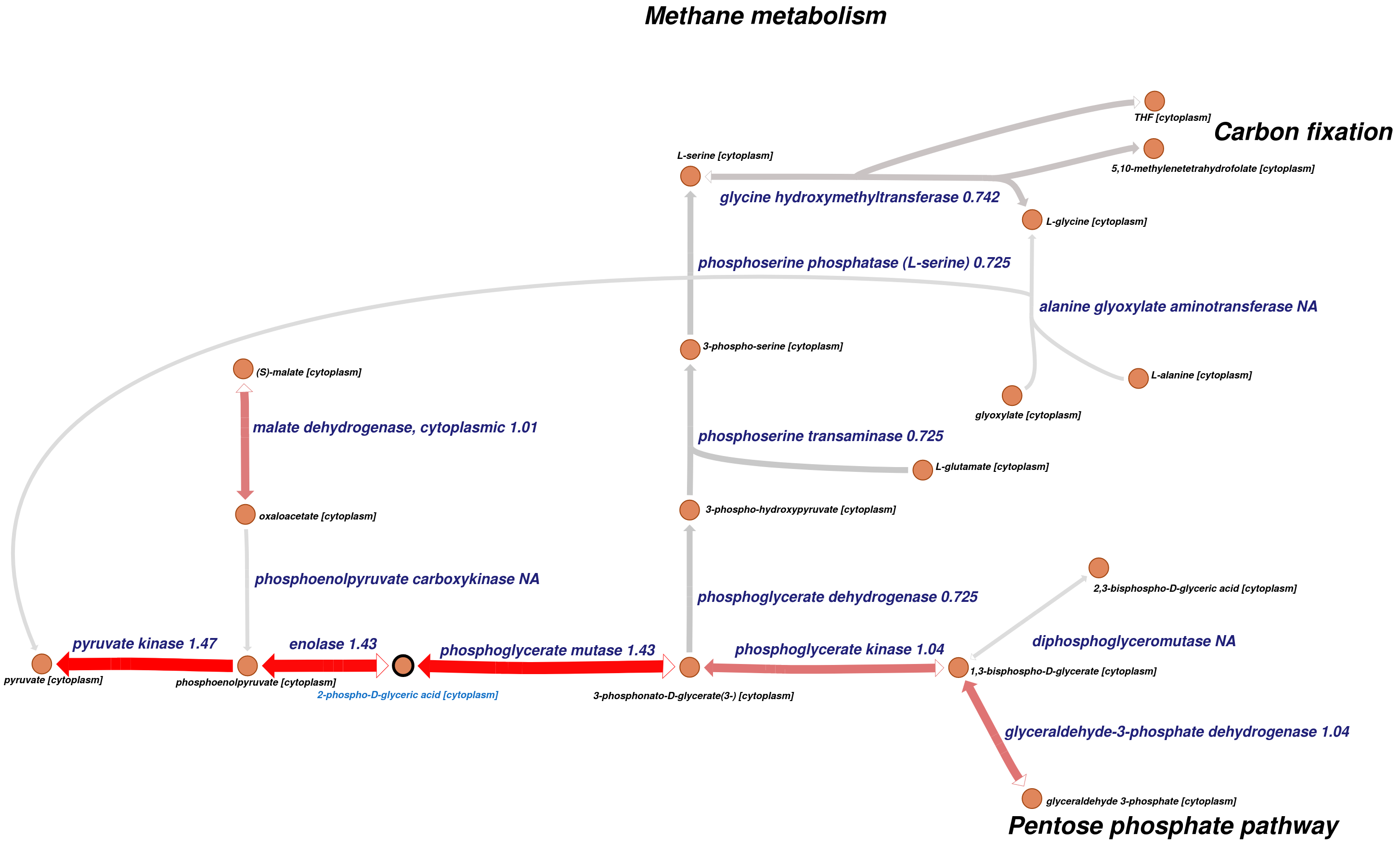

Supplement: S11 Fig — The flux ratios between treated and control model FBA solutions are represented. Edges' thickness and color are a function of the respective ratio values. Only a fraction of KEGG’s reference pathway is present in yeast. (TIF) [file pone.0223909.s011.tif]

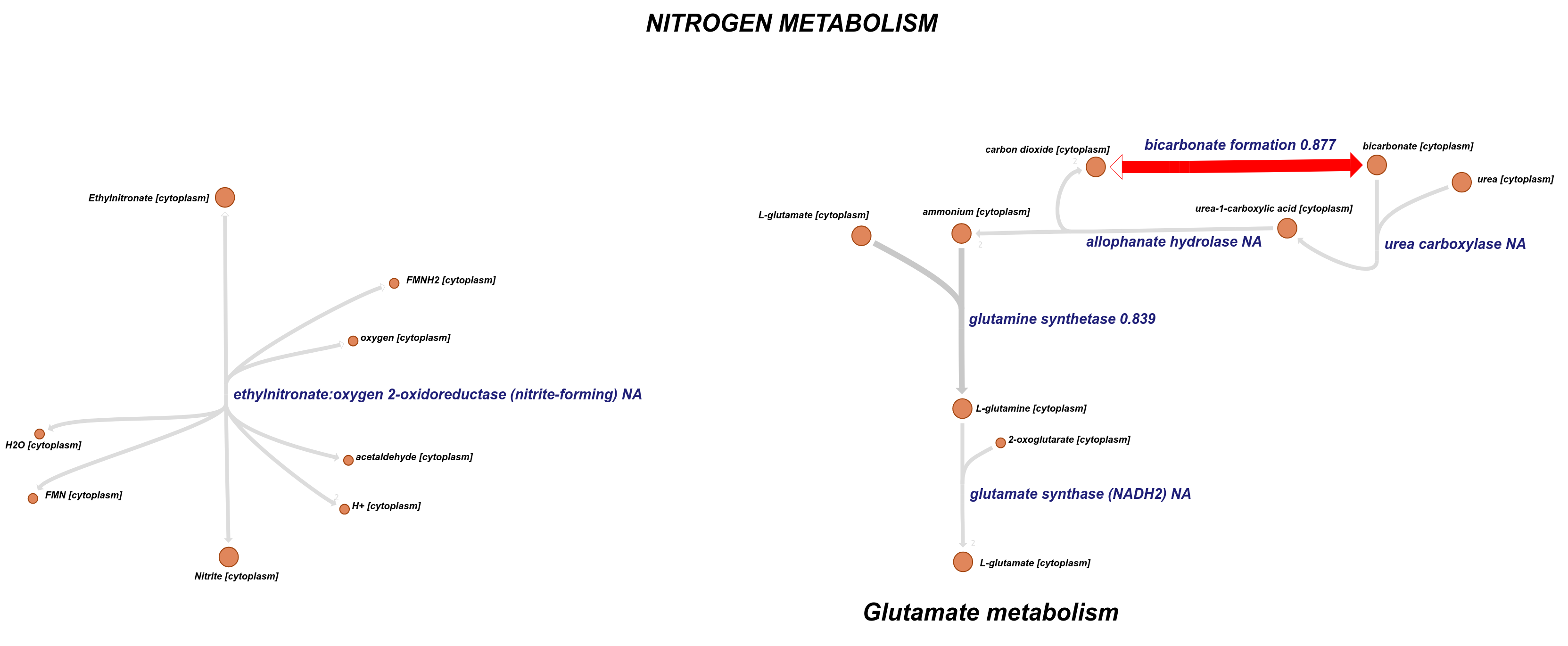

Supplement: S12 Fig — The flux ratios between treated and control model FBA solutions are represented. Edges' thickness and color are a function of the respective ratio values. (TIF) [file pone.0223909.s012.tif]
